# The colibactin-producing *Escherichia coli* alters the tumor microenvironment to immunosuppressive lipid overload facilitating colorectal cancer progression and chemoresistance

**DOI:** 10.1080/19490976.2024.2320291

**Published:** 2024-02-28

**Authors:** Nilmara de Oliveira Alves, Guillaume Dalmasso, Darja Nikitina, Amaury Vaysse, Richard Ruez, Lea Ledoux, Thierry Pedron, Emma Bergsten, Olivier Boulard, Lora Autier, Sofian Allam, Laurence Motreff, Pierre Sauvanet, Diane Letourneur, Pragya Kashyap, Johan Gagnière, Denis Pezet, Catherine Godfraind, Michel Salzet, Emmanuel Lemichez, Mathilde Bonnet, Imène Najjar, Christophe Malabat, Marc Monot, Denis Mestivier, Nicolas Barnich, Pankaj Yadav, Isabelle Fournier, Sean Kennedy, Amel Mettouchi, Richard Bonnet, Iradj Sobhani, Mathias Chamaillard

**Affiliations:** a ONCOLille, INSERM, Phycell, University of Lille, Lille, France; b Microbes, Intestin, Inflammation et Susceptibilité de l’Hôte (M2iSH), Centre de Recherche en Nutrition Humaine Auvergne, Université Clermont Auvergne, Clermont-Ferrand, France; c CNRS, Institute Pasteur, Paris, France; d Laboratory of Clinical and Molecular Gastroenterology, Institute for Digestive Research, Lithuanian University of Health Sciences, Kaunas, Lithuania; e Institut Pasteur, Université Paris Cité, Bioinformatics and Biostatistics Hub, Plate-Forme Technologique Biomics, Paris, France; f Réponse Inflammatoire et Spectrométrie de Masse-PRISM, University of Lille, Lille, France; g Institut Pasteur, Inserm, Paris, France; h Institut Pasteur, Université Paris Cité, Paris, France; i Department of Bioscience & Bioengineering, Indian Institute of Technology, Jodhpur, Rajasthan, India; j Université Paris Est Créteil, Créteil, France; k Service de Gastroentérologie CHU Henri Mondor, Assistance Publique des Hôpitaux de Paris-APHP, Créteil, France

**Keywords:** Colibactin-producing *Escherichia coli*, land’s cycle, lipid droplet, right-sided colorectal cancer

## Abstract

Intratumoral bacteria flexibly contribute to cellular and molecular tumor heterogeneity for supporting cancer recurrence through poorly understood mechanisms. Using spatial metabolomic profiling technologies and 16SrRNA sequencing, we herein report that right-sided colorectal tumors are predominantly populated with Colibactin-producing *Escherichia coli* (CoPEC) that are locally establishing a high-glycerophospholipid microenvironment with lowered immunogenicity. It coincided with a reduced infiltration of CD8^+^ T lymphocytes that produce the cytotoxic cytokines IFN-γ where invading bacteria have been geolocated. Mechanistically, the accumulation of lipid droplets in infected cancer cells relied on the production of colibactin as a measure to limit genotoxic stress to some extent. Such heightened phosphatidylcholine remodeling by the enzyme of the Land’s cycle supplied CoPEC-infected cancer cells with sufficient energy for sustaining cell survival in response to chemotherapies. This accords with the lowered overall survival of colorectal patients at stage III-IV who were colonized by CoPEC when compared to patients at stage I-II. Accordingly, the sensitivity of CoPEC-infected cancer cells to chemotherapies was restored upon treatment with an acyl-CoA synthetase inhibitor. By contrast, such metabolic dysregulation leading to chemoresistance was not observed in human colon cancer cells that were infected with the mutant strain that did not produce colibactin (11G5*∆ClbQ*). This work revealed that CoPEC locally supports an energy trade-off lipid overload within tumors for lowering tumor immunogenicity. This may pave the way for improving chemoresistance and subsequently outcome of CRC patients who are colonized by CoPEC.

## Introduction

Colorectal cancer (CRC) is the third most common form of malignancy and the second leading cause of cancer-related death worldwide.^1^ Patients with right-sided CRC have a worse prognosis than left-sided CRC and it has already been reported that they do not respond well to conventional chemotherapies.^2^ Chemotherapy drugs used to treat CRC primarily include oxaliplatin and cytotoxic drugs that inhibit the enzyme activity of thymidylate synthase 5-Fluorouracil. Whereas most patients with advanced CRC are primarily responsive to first-line chemotherapies, the 5-year survival rate is lower than 10% as a consequence of acquired chemoresistance. Only recently has been established that tumor-type features of the intratumoral microbiota are closely associated with resistance to chemotherapies and tumor recurrence.^3,4^ Specifically, some bacteria of often very low biomass may populate specific niches within the metastatic tumor microenvironment for impeding immune surveillance, such as *Fusobacterium nucleatum*.^5^ The use of patient-derived xenograft models revealed that intratumoral microniches with *Fusobacterium nucleatum* are maintained even in distant metastases.^6^ Consequently, the intracellular presence of *Fusobacterium nucleatum* in CRC leads to the acquisition of chemoresistance through the modulation of autophagy.^4^

Colibactin is a secondary metabolite encoded by the pks pathogenicity island of certain *Escherichia coli* strains that preferentially colonize the right-sided colon.^7,8^ Bonnet et al. reported a poor prognosis outcome is related to the colonization by Colibactin-producing *Escherichia coli* (CoPEC),^9^ which is detected in about 50–60% of human CRC biopsies compared to ~ 20% of patients with diverticulosis.^10,11^ This suggests that CoPEC may contribute to the recurrence of CRC. Accordingly, it has been established that Colibactin induces alteration of p53 SUMOylation^8^ and double-strand breaks^12^ as well as generates DNA adducts^13^ and genomic aberrations with an increased mutational load.^14,15^ This led to the identification of a specific DNA damage signature with an AT-rich hexameric sequence motif in tumors of patients that have been colonized by CoPEC strains.^16^ Accordingly, colon tumorigenesis is accelerated in APC^Min/+^ mice that are colonized by CoPEC.^9,17^ Another work evidenced that organoids that recovered from short-term infection with CoPEC show characteristics of CRC cells including enhanced proliferation, Wnt-independence, and impaired differentiation that are often achieved by mutations in the gene encoding for Adenomatous polyposis coli (APC).^18^ In APC^Min/+^ mice, colon tumorigenesis is accelerated following colonization by CoPEC through modulation of the autophagic pathway in intestinal epithelial cells.^15^ However, the potential effect of Colibactin on chemotherapy has not yet been examined in CRC.

Herein, we identified that colonization by CoPEC is an unfavorable prognostic factor in a subtype of poorly immunogenic right-sided CRC tumors that are defined as consensus molecular subtype 3 with a lipid overload. Specifically, spatially esolved metabolomics applied to tumors that are colonized by CoPEC revealed a significant intratumoral deposit of glycerophospholipids in areas that are populated by bacteria. Taken together, our findings clarify how Colibactin may establish tumor resistance to chemotherapies. This provides unique insights for fostering the development of therapies targeting the oncogenic-driven lipid reprogramming that is induced by CoPEC.

## Results

### Right-sided CRC tumors that are colonized by CoPEC are associated with cancer recurrence and a distinct microbiome composition

**Figure 1.**
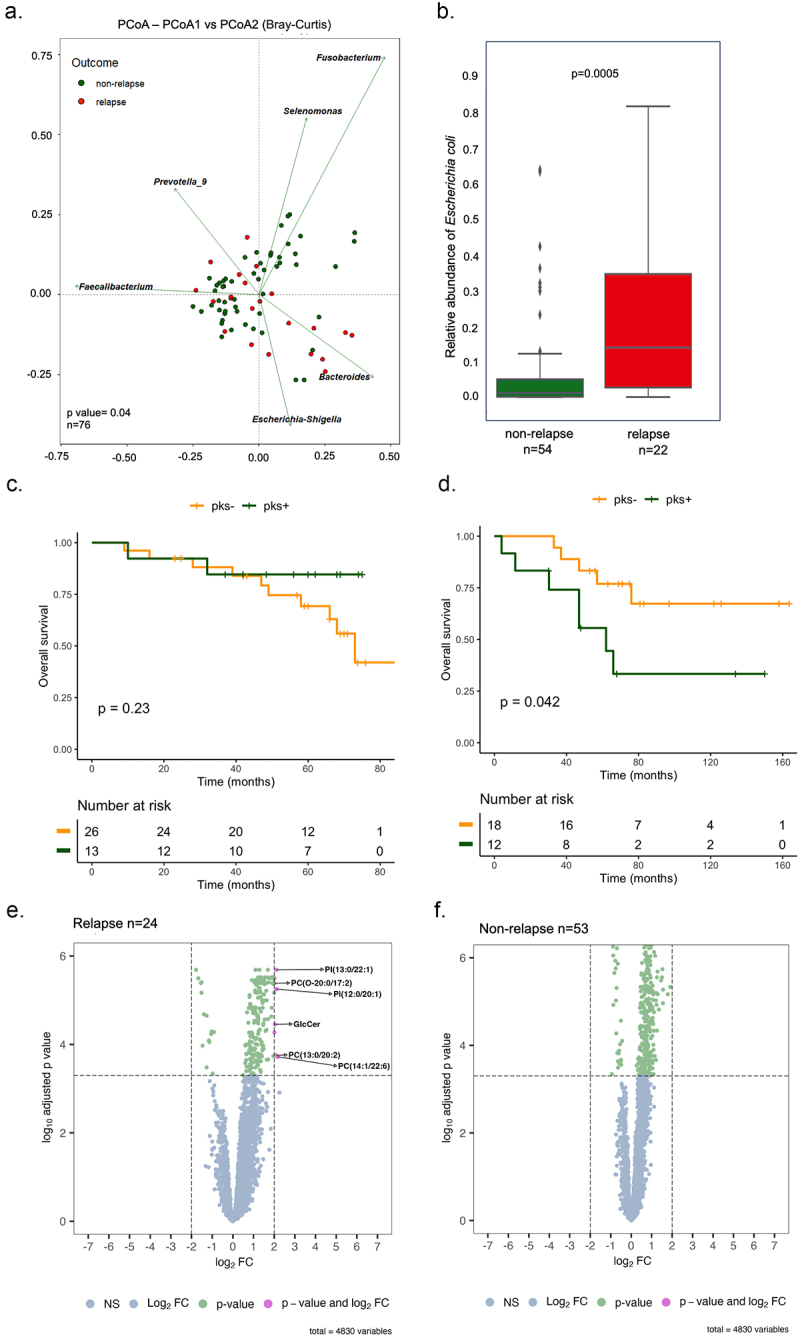
Patients with right-sided colorectal cancer exhibit distinct bacterial profiles and showed poor survival in patients colonized by Colibactin. (a) Principal coordinate analysis (PCoA) with Bray-Curtis distances matrix between non-relapse and relapse right-sided CRC patients. (b) Box plot with the relative abundance of *Escherichia coli*. The significance p-value was calculated using the Wilcoxon rank sums test. (c) Kaplan-Meier analysis from patients colonized by *Escherichia coli* harboring the pks island at stage I-II. (d) Kaplan-Meier analysis from patients colonized by *Escherichia coli* harboring the pks island at stage III-IV. (e) and (f) volcano plots of differentially expressed metabolites between tumor tissue and tumor-adjacent stroma in two groups: relapse (24 tumors versus 21 tumors-adjacent stroma) and non-relapse (53 tumors versus 51 tumors-adjacent stroma), respectively. Dashed lines indicated the following significance threshold: 2.0 > log_2_ fold change < -2.0 and FDR-adjusted p-value <0.0005.

Microbiome profiling revealed that right-sided colon tumors are associated with a dense community of bacteria encased in a likely complex matrix that contacts the colon epithelial cells.^18^ This led us to examine whether the composition of intratumoral microbiota could be linked to right-sided CRC recurrence that is mainly attributed to chemoresistance. To this end, we performed 16S rRNA gene sequencing on tumor samples of 76 patients with right-sided CRC from two independent cohorts among which 22 patients were in relapse. The sequencing data were compared to the one from 11 patients with left-sided CRC, among which 3 patients were in relapse. We investigated the biodiversity of a total 3511 of amplicon sequence variants (ASVs) that were produced by the DADA2 method after filtering. The normalized mean count of genus and species was calculated separately based on the area under the receiver operating characteristic (AUROC) curve score. Our qualitative analysis shows a higher mean count in the relapsing group compared to non-relapsing at both genus and species levels (Supplementary Figure S1A and S1B). Following confirmation of normality through the Shapiro-Wilk normality test, a Mann-Whitney t-test was applied to evaluate the potential impact of age distributions and gender differences between patients in relapse or not. As what was observed for age on panel C of the supplementary Figure S1 (p-value = 0.14), an examination of sex distribution between the two groups, conducted through Fisher’s test, did not yield a statistically significant difference (p-value = 0.8046) (Supplementary Figure S1D). Even though no significant differences were noticed with several estimates of alpha diversity between relapse and non-relapse groups from either right- or left-sided CRC patients (Supplementary Figure S1E). However, when comparing bacterial diversity (Simpson and Shannon indices) between right- and left-sided colon samples, statistically significant distinctions emerged for both relapse and non-relapse patients (Supplementary Figure S1F and G). In addition, the beta diversity based on Bray Curtis dissimilarity was found to be significantly lowered in relapsing patients when compared to patients in remission (Permanova *p* = 0.045 at genus level, Figure 1A). Among the differentially abundant genera, we found that the tumors from patients in remission were significantly enriched in five species, which are *Bacteroides plebeius*, *Colidextribacter massiliensis*, *Dialister pneumosintes*, *Fusobacterium nucleatum* and *Lactococcus lactis* (FDR-adjusted p-value <0.05, Supplementary Table S1). By contrast, the genus *Escherichia-Shigella* was the only bacterial population that was dominant in most primary tumors from relapsing patients with right-sided CRC cancer (Supplementary Table S1). Accordingly, the species *Escherichia coli* was the most enriched in recurrent right-sided CRC tissues as compared to those from patients without relapse (*p* = 0.0005, FDR-adjusted p-value = 0.044, Figure 1B). A beta-diversity analysis identified variations in the composition of bacteria between the right- and left-sided tumors (Supplementary Table S2 and S3).

We further investigated whether the presence of *pks* within the tumor could influence survival outcomes in patients with stage I-II as well as stage III-IV. The detection of the genomic island *pks* was validated by two different PCR-based methods on DNA that were extracted from right- and left-sided CRC tissue. This revealed that right-sided CRC patients colonized by CoPEC showed lower overall survival at stage III-IV(*p* = 0.042), but not those patients from stage I-II (*p* = 0.23) (Figure 1C,D).

Given the association between loss of function mutation in TP53 and CoPEC, we explored the possibility that such mutation may contribute at least partially to disease relapse. Whole exome sequencing revealed a similar survival rate between patients with right-sided tumors bearing or not loss of function mutation in TP53 (Supplementary Figure S2). Among the right-sided CRC cases with TP53 mutations, we found that 15.38% involved recurrence and 30.77% exhibited were positive for pks (Supplementary Figure S3A). By contrast, half of the patients with left-sided CRC and TP53 mutations experienced tumor recurrence, even though a minimum of half did not show colonization by CoPEC (50% pks^−^, 37.5% pks^+^ and 12.5% not analyzed, Supplementary Figure S3B). Intriguingly, a higher number of differences were observed in the non-relapse group (including the *Escherichia-Shigella* genera) compared to the relapse group when comparing left- and right-sided samples. Nevertheless, drawing definitive conclusions from the analysis in left-sided CRC proves challenging due to the limited number of samples in the left-sided patient group. In summary, a notable bacterial distinction exists between right- and left-sided tumors independently of TP53. This difference is less pronounced for patients in relapse. This is in agreement with the worst prognosis that has been documented in patients who are colonized by CoPEC.^9^

### CoPEC-modulated disease recurrence is linked to alterations in lipid metabolism

Given that *Escherichia coli* was more abundant in the group of relapsing patients’, we asked the question of whether it may be linked to a specific metabolic profile by applying a high spectral resolution of the 7T-MALDI-FTICR to 77 right-sided CRC tumoral specimens. The discrimination of different tissue regions: non-tumoral, stroma and tumor was performed by an expert pathologist. Based on the MALDI-HE overlay image, the tumor region and tumor-adjacent stroma were delimited and the metabolites quantification was carried out in the respective regions. As shown in Figure 1E,F, a significant increase in the lipid intensity was observed in the tumor region compared to tumor-adjacent stroma only in the relapse group. Among the eight significantly expressed metabolites, six metabolites (PC(13:0/20:2(11Z,14Z)); PC(O-20:0/17:2(9Z,12Z)); PC(14:1(9Z)/22:6(4Z,7Z,10Z,13Z,16Z,19Z)); PI(12:0/20:1(11Z)); PI(16:0/18:2(9Z,12Z)); PI(13:0/22:1(11Z))) were identified in the glycerophospholipid class, one (GlcCer(d18:1(8Z)/24:0(2OH[R]))) was identified as sphingolipid and only one was not annotated (Supplementary Table S4). Accordingly, bulk RNA-seq analysis revealed that more than 50% of tumors colonized by CoPEC were classified as CMS3 that are characterized by metabolic dysregulation with higher activity in glutaminolysis and lipogenesis. Collectively, these results could indicate that tumor cells can reshape the microenvironment by lipid metabolism reprogramming supported by abundant colonization by *Escherichia coli.*

### CoPEC-modulated disease recurrence is linked to alterations in lipid metabolism

**Figure 2.**
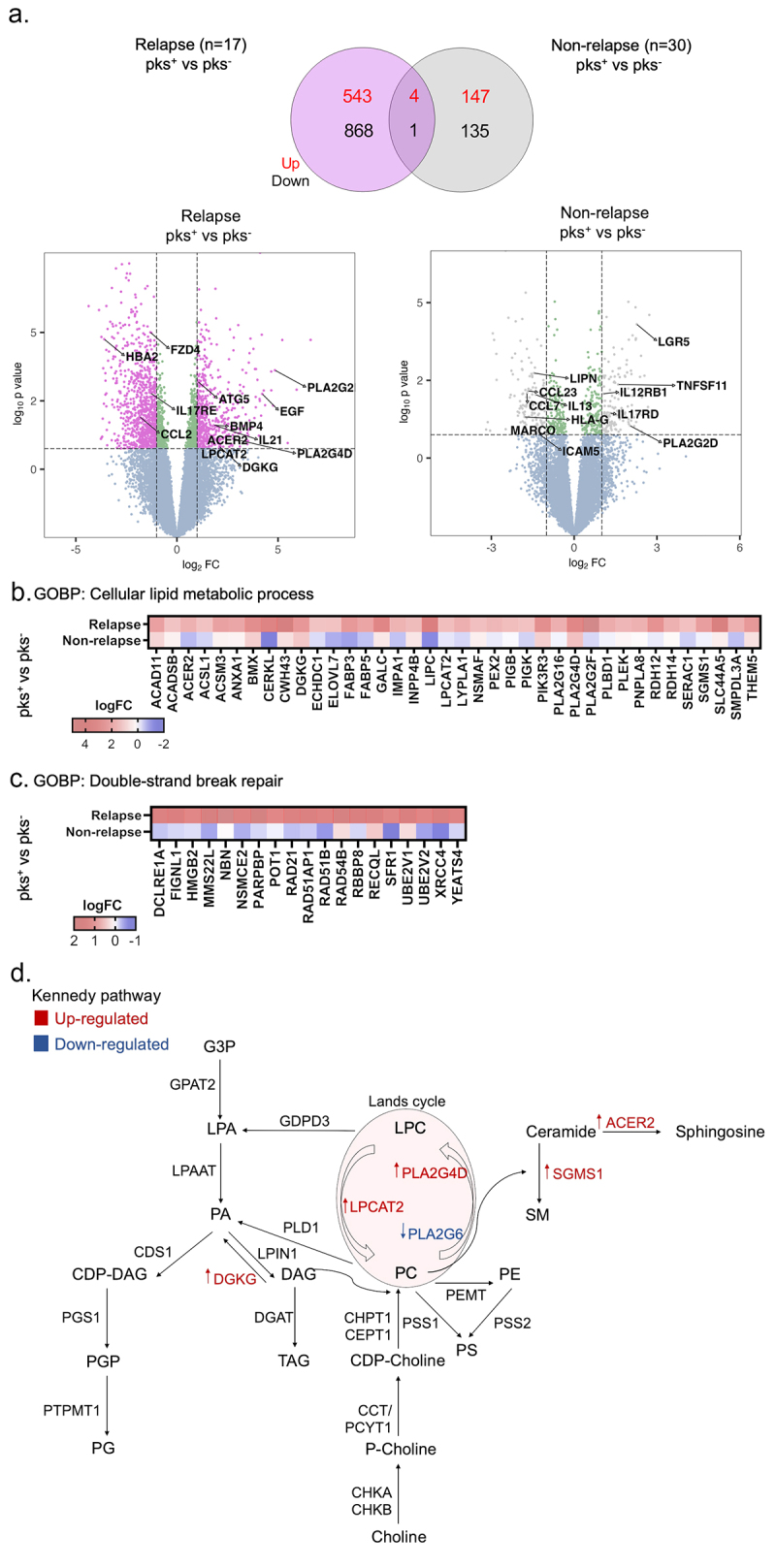
Right-sided colorectal cancer patients colonized by CoPEC with relapse reveal enrichment of genes associated with lipid metabolism and DNA double-strand break. (a) Volcano plots and venn diagram of differentially expressed genes in response to pks^+^ versus – pks^−^ tumors from relapse (*n* = 17) and non-relapse groups (*n* = 30). Dashed lines indicated the following significance threshold: 1.0 > log_2_ fold change < -1.0 and p-value <0.05. (b) and (c) heatmap showing logFC of genes associated with cellular lipid metabolic process and double-strand break repair (FUMA-GO biological process) in response to pks^+^ versus -pks^−^ tumors from relapse and non-relapse groups. (c) Schema showing genes involved in the biosynthesis of the lipid in both the Kennedy pathway (De novo synthesis) and lands cycle. Highlighted genes in response to pks^+^ versus -pks^−^ tumors from the relapse group. Red: up-regulated and blue: down-regulated.

The greater abundance of bacteria related to *Escherichia coli* in relapse patients led us to explore the transcriptomic landscape in the different groups concerning patients colonized by pks-harboring *Escherichia coli* (pks^+^) or non (pks^−^) with relapse as well as those without recurrence (non-relapse group). Differential gene expression analysis between pks^+^ and – pks^−^ tumors from patients with relapse revealed 547 up- and 869 down-regulated genes. On the other hand, pks^+^ and – pks^−^ tumors from patients without recurrence showed 151 up- and 136 down-regulated genes (Figure 2A). By performing functional mapping and annotation (FUMA), biological processes gene ontology (GO) term analysis was presented in Supplementary Figure S4, among which cell division, cell cycle process, cellular response to stress, phospholipid and glycerophospholipid process, DNA damage stimulus and others were closely associated with Colibactin in CRC progression. For instance, up-regulated genes in pks^+^ tumors from the relapse group were enriched in the cellular lipid metabolic process and double-strand break repair (Figure 2B,C). Especially, mRNA levels associated with the regulation of lipid metabolism were overrepresented. Among them, diacylglycerol kinase gamma (DGKG), a gene that encodes an enzyme that generates phosphatidic acid (PA) by catalyzing the phosphorylation of diacylglycerol (DAG) was up-regulated in tumors colonized by Colibactin in the relapse group. We also observed that Lysophosphatidylcholine acyltransferase 2 (LPCAT2), which plays a role in phospholipid metabolism in the conversion of lysophosphatidylcholine (LPC) to phosphatidylcholine (PC) in the presence of acyl-CoA was increased in these same patients. In addition, different changes in the phospholipase A2 enzyme family genes, that catalyze the hydrolysis of the sn-2 position of membrane glycerophospholipids generating free fatty acids and lysophospholipids were identified. For example, the expression of the genes encoding for PLA2G16, PLA2G4D and PLA2G2F were up-regulated in tumors colonized by CoPEC. In contrast, transcript level of PLA2G6 was down-regulated in this group. Interestingly, these alterations were accompanied by overexpression of mRNAs implicated in the ceramide metabolism such as alkaline ceramidase 2 (ACER2) and sphingomyelin synthase 1 (SGMS1). The ceramidase ACER2 hydrolyzes long-chain ceramides to generate sphingosine, while SGMS1 catalyzes in the forward reaction of transferring the phosphocholine head group of PC onto ceramide to form sphingomyelin (SM) (Figure 2D). By contrast, RNA-seq analysis of left-sided CRC tumors failed to detect changes in the expression of the aforementioned genes if any (Supplementary Figure S5). Together, these lipid enzyme alterations encouraged us to investigate if Colibactin-producing bacteria may be involved in the lipid metabolic reprogramming of tumor cells.

### Spatially resolved metabolomic approach unveils bacterial regions that are highly metabolically active in response to CoPEC colonization

**Figure 3.**
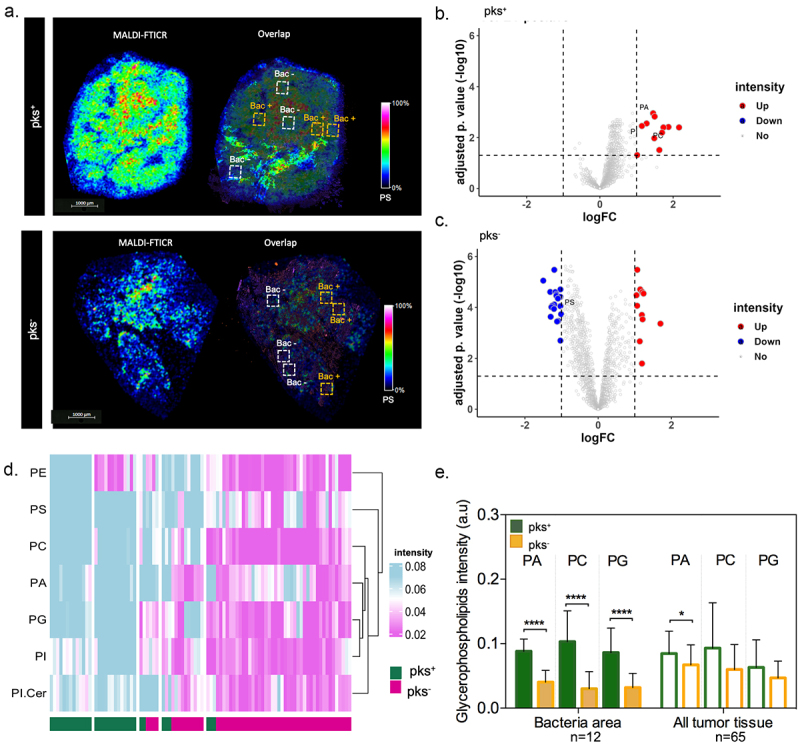
Spatial metabolomics revealed an increase in glycerophospholipid in tumor-associated bacterial microniches mainly in patients colonized by colibactin-producing *Escherichia coli* (CoPEC). (a) Representative images of MALDI-FTICR and overlap with FISH (bacteria stained with general rRNA probe EUB338 conjugated to Alexa 555 - orange) from pks^+^ and – pks^−^ tumors. (b) and (c) volcano plot displaying metabolomic analysis from pks^+^ (ROI with bacteria versus ROI without bacteria) and pks^−^ (ROI with bacteria versus ROI without bacteria) patients, respectively. Root means square normalization was applied, adjusted p. value (FDR correction) < 0.05, and log_2_FC >1 or < -1 were considered. (d) Heatmap showing differentially expressed lipids in the ROI with bacteria between pks^+^ and – pks^−^ tumors. (e) Graphic showing the difference in metabolite expression in bacteria area (*n* = 12) and all tumor tissue (*n* = 65).

The response of gene expression associated with lipid metabolism in pks^+^-infected tumors led us to study the potential consequences of metabolic reprogramming of areas that are invaded by bacteria. To this end, spatially resolved metabolomics was applied on the 12 right-sided CRC tissue samples using a high spectral resolution of the 7T-MALDI-FTICR. By using *in situ* hybridization (FISH) imaging, we visually confirmed the heterogeneous geolocation of bacterial microniches in right-sided CRC tissues. On each tissue, 3–13 specific regions of interest (ROI) were selected according to the FISH image (Figure 3A). This resulted in a total of 90 ROI with bacteria microniches and 86 ROI without bacteria. Here, we detected 12 up-regulated metabolites in ROI with bacteria compared to ROI without bacteria in pks^+^ tumors (Figure 3B). Moreover, comparisons between ROI with and without bacteria in pks^−^ tumors revealed 20 metabolites down and 11 over-expressed (Figure 3C). Specifically, 11 metabolites were found up-regulated only in patients colonized by pks^+^ such as Benzenoids (*n* = 1), Fatty Acyls (*n* = 1), Glycerophospholipids (*n* = 3), Organic acids (*n* = 1), Organoheterocyclic (*n* = 2), Sphingolipids (*n* = 1) and Sterol Lipids (*n* = 2)). In addition, metabolomic differential expression in ROI with bacteria microniches revealed a predominance of lipids (approximately 40%) in the pks^+^ compared with the pks^−^ patients’ groups. Among those lipids identified, approximately 82% belong to the glycerophospholipids subclass such as phosphatidic acid (PA), PC, phosphatidylinositol (PI), phosphatidylethanolamine (PE), phosphatidylserine (PS), phosphatidylglycerol (PG) and others (Figure 3D). Subsequently, we performed a comparative analysis of metabolite intensity for the same m/z in all tumor tissue and the areas with bacteria. This showed that most of the metabolic heterogeneity is explained by the presence of microniches populated with bacteria (Figure 3E). Expectedly, such a difference was not observed in left-sided tumor samples (Supplementary Figure S6A,B). However, a greater abundance of such lipids was also noticed in right-sided CRC tumors when compared to those that were located on the descending colon (Supplementary Figure S6C,D). Given that disturbances in the gut microbial profiles/communities are associated with heightened *de novo* lipogenesis, this led us to suggest that CoPEC may be responsible for metabolic dysregulation that promotes carcinogenesis in the ascending colon.

### Lipid droplet accumulates in colon cancer cells that are infected by CoPEC

**Figure 4.**
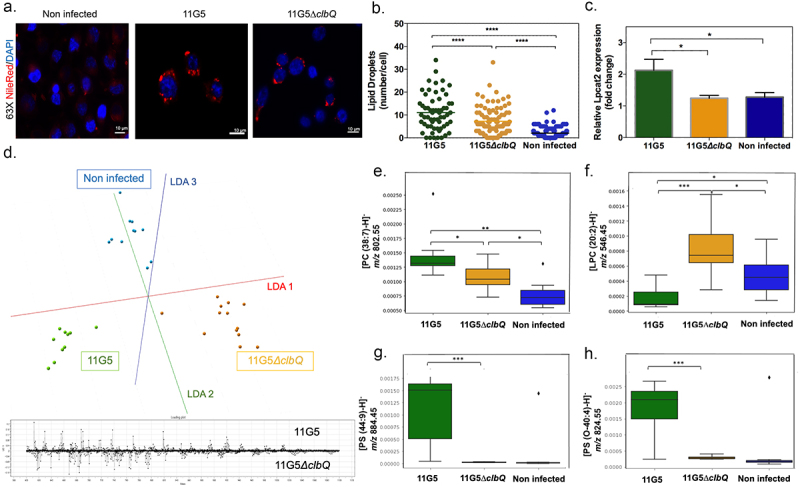
Bacterial colibactin leads to lipid droplet accumulation in human colon cancer cells. (a) Representative image of Nile red staining (×63 magnification, scale bar = 10 µm) using HCT116 cells. Nuclei (DAPI – blue), lipid droplet (red). (b) Lipid droplet quantification (300 cells) under different bacterial infections: CoPEC 11G5 strain, isogenic mutant 11G5*ΔclbQ* strain and negative control (NC, non-infected). (c) MC38 cells were infected with the 11G5 strain or the 111G5*ΔclbQ* and were analyzed 5 days post-infection. Lpcat2 mRNA levels from MC38 cells were quantified using qRT-PCR. (d) The built PCA-LDA classification model is based on three groups: HCT116 cells infected with CoPEC strain (11G5), a mutant strain that does not produce CoPEC (11G5∆clbQ) and non-infected (negative control). Representative m/z chromatograms. (e – h) graphs with the intensity of PC, LPC and PS differentially expressed under the conditions studied.

To assess whether colibactin may directly trigger lipid overload, the human colon carcinoma HCT116 cells were infected with CoPEC strain (11G5) isolated from a patient CRC or a mutant strain that does not produce Colibactin (11G5*∆clbQ*).^19^ In agreement with our spatial metabolomic analysis, CoPEC infection leads to lipid droplet accumulation and an increase in LPCAT2 levels (Figure 4A–C). SpiderMass technology was then applied to define which glycerophospholipids contribute to the discrimination between 11G5-, 11G5*∆clbQ*-infected and non-infected HCT116 cells. This led us to identify several differentially abundant glycerophospholipids such as PC, PS, PE and PI and decrease of LPC in response to CoPEC infection (Figure 4D–H). By contrast to what was observed in response to cellular stress by chemotherapy, the level of ceramide was significantly reduced in response to CoPEC (Supplementary Table S5). Similar results were obtained with the CRC carcinoma cell line MC38 (Supplementary Table S6). Together, the CoPEC-induced lipid droplet accumulation was preceded by a remodeling of these results encouraged us to continue investigating the mechanisms of how tumor-associated CoPEC can promote lipid reprogramming.

### CoPEC-induced lipid droplet accumulation and chemoresistance are preceded by elevated levels of reactive oxygen species (ROS)

**Figure 5.**
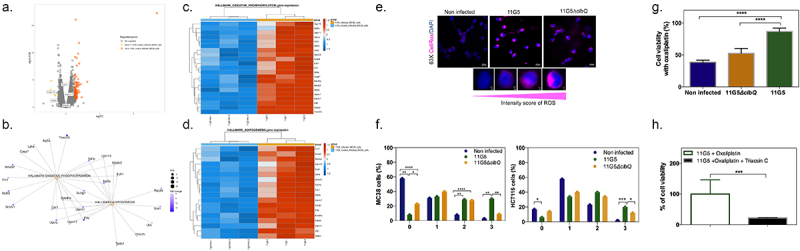
Chemoresistance in CRC cells is preceded by elevated levels of reactive oxygen species after CoPEC colonization. (a) Volcano plot representation of differentially expressed genes between MC38 infected with a mutant strain that does not produce CoPEC (11G5∆clbQ) and CoPEC strain (11G5). (b-d) representation of the enrichment of genes involved in both oxidative phosphorylation and adipogenesis. (e) Representative image of CellRox staining for intracellular reactive oxygen species in HCT116 infected with CoPEC strain (11G5), a mutant strain that does not produce CoPEC (11G5∆clbQ) and non-infected (negative control). Fluorescent images were captured after 4 h of treatment (3 h of infection +1 h pos-infection) under MOI 10. (f) A semi-quantitative assessment of the percentage of ROS in 300 cells was evaluated using different scores from 0 to 3 using MC38 and HCT116 cells. Data are expressed by the percentage of cells in each score and group (two independent experiments and duplicates for each experiment). * *p* < 0.05, ** *p* < 0.01, *** *p* < 0.001 and *** *p* < 0.0001. (G) MC38 cells were infected with CoPEC strain (11G5), a mutant strain that does not produce CoPEC (11G5*∆clbQ*) and non-infected supplemented with oxaliplatin (20 μg/mL). Non-infected cells without oxaliplatin were used to represent 100% viability. Values represent means ± SEM. (H) MC38 cells were infected with CoPEC strain (11G5) and 3-week post-infection cells were used supplemented with oxaliplatin (5 μg/mL) in the absence or presence of triacsin C (10 µM). 11G5-infected cells without oxaliplatin were used to represent 100% viability. Values represent means ± SEM. Cell viability was assessed using WST-1 assay reagent – cell proliferation assay.

We next aimed at characterizing the early stress response of CRC cancer cells to CoPEC that precedes lipid droplet accumulation by performing RNA-seq analysis. Applying the edgeR method revealed a total of 98 differentially expressed genes in response to CoPEC when compared to its mutant that is unable to produce colibactin (*p* < 0.0001). Among those, 94 were significantly up-regulated as displayed with a Volcano plot (Figure 5A). Over-representation analysis revealed that several differentially expressed genes were functionally related to processes that are related to both oxidative phosphorylation and adipogenesis, including *Cyc1* which encodes for a subunit of the cytochrome bc1 complex and *Suclg1* that encodes for a Succinate-CoA Ligase GDP/ADP-Forming Subunit Alpha (Figure 5B–D). These results suggested that CoPEC may locally promote the accumulation of lipid droplets through the induction of hypoxia and extracellular acidosis. Since lipid droplet production can act as switches in response to imbalances in energy metabolism and redox homeostasis,^20^ we next investigated the earlier accumulation of ROS in HCT116 cells and another model of CRC cancer cells that is the syngeneic MC38 cell line. As shown in Figure 5E,F, a semi-quantitative assessment of the percentage of cells with CellRox was performed (score 0 to 3, see Material and Methods section) and a notable increase in cells with high fluorescence (as determined by a score of 3) was observed after CoPEC infection. Given that elevated ROS may result in immunogenic cell death, we hypothesized that the accumulation of lipid droplets may be a protective mechanism that curtails the efficacy of anticancer drugs. This led us to evaluate whether CoPEC-induced ROS formation could affect the sensitivity of CRC cells to oxaliplatin which is a third-generation diaminocyclohexane-containing platinum compound. As shown in Figure 5G, 11G5-infected cells were more resistant to oxaliplatin compared to 11G5Δpks-infected cells and non-infected cells. Since we know that lipid droplet formation increases after CoPEC infection, we used Triacsin C, which is a consequent lipid droplet blocker by inhibiting of long-chain fatty acyl CoA synthetase. Accordingly, CoPEC infection in the presence of Triacsin C remarkably lowered resistance to oxaliplatin of MC38 (Figure 5H). This is in agreement with previous studies showing that the level of enzyme supporting PC synthesis is enhanced in oxaliplatin-resistant cells compared to untreated parental cells.^21^

### CoPEC-induced lipid droplet accumulation impairs the immunogenicity of right-sided CRC

**Figure 6.**
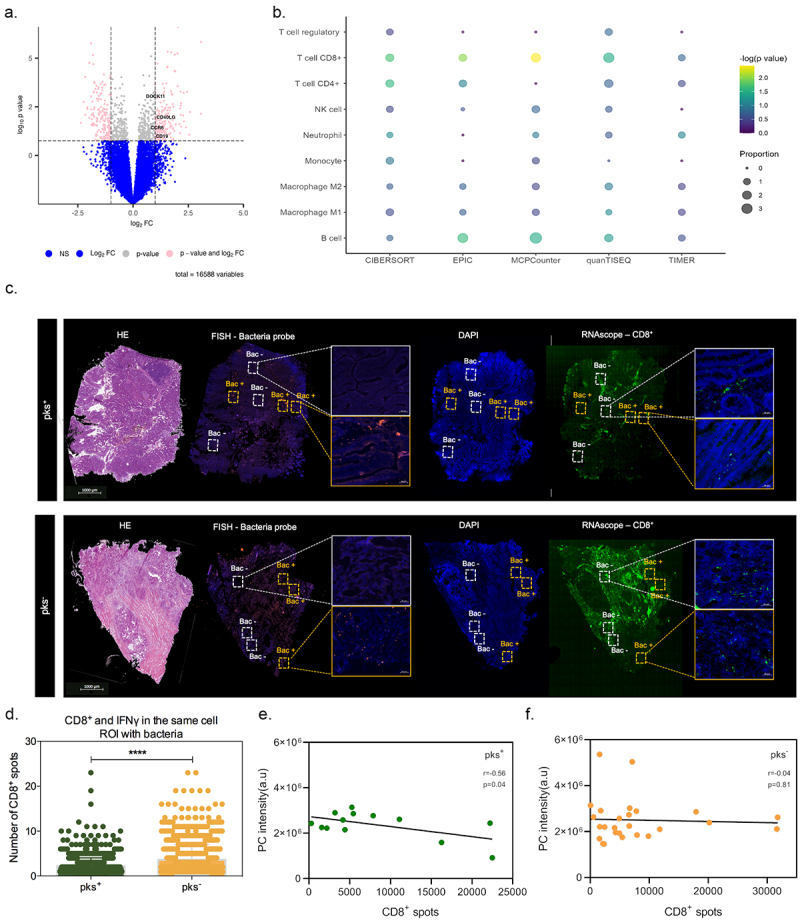
Bacterial microniches are poorly infiltrated with IFNγ-producing CD8^+^ T-cells in right-sided CRC patients colonized by CoPEC. (A) Volcano plot representation of differentially expressed genes between CoPEC-positive and -negative patients. Dashed lines indicated the following significance threshold: 1.0 > log_2_ fold change < -1.0 and p-value <0.05. (B) Bubble plot showing the proportional difference of immune cells between pks^+^ and – pks^−^ patients based on the following computational methods: CIBERSORT, EPIC, MCP-counter, quanTiseq and TIMER. (C) Representative images of H&E, *in situ* hybridization (FISH – bacteria stained with general rRNA probe EUB338 conjugated to Alexa 555 - orange) and the nuclear DNA stained with DAPI (blue)), RNAscope (hs-CD8A (560391-C3, Opal 520 - green) from pks^+^ and – pks^−^ tumors. White square: ROI without bacteria and orange square: ROI with bacteria. (D) Quantification of CD8^+^ T-cells and IFNγ in the same cells in the ROI with bacteria from pks^+^ and – pks^−^ tumors. **** *p* < 0.0001. (E) and (F) Pearson correlation between CD8^+^ T-cell spots detected by the RNAscope and phosphatidylcholine (PC) identified by the MALDI-FTICR in pks^+^ and – pks^−^ tumors, respectively.

The aforementioned results led us to postulate that the formation of lipid droplets may lower immunogenicity by supporting acidosis-driven epithelial-to-mesenchymal transition. Accordingly, bulk RNAseq analysis from tumor tissues unveiled a down-regulation of several genes that are related to B cell activation in CoPEC-positive tumors, such as CD19, CCR6, CD40LG and DOCK11 (Figure 6A). Accordingly, a significant enrichment of cytotoxic CD8^+^ T-cells and B-cells proportion was observed in CoPEC-positive tumors when using the web-accessible TIMER2.0 algorithm (Figure 6B). This led us to determine whether there is a difference in CD8^+^ T-cell infiltrates in intra- and inter-tumor annotated as ROI with or without bacteria. Accordingly, RNAscope-FISH overlay images revealed that bacteria microniches are characterized by immunosuppressive effect with a decrease of tumor-infiltrating CD8^+^ T-cells, mainly in patients colonized by CoPEC (Figure 6C). Analyzing specific CD8^+^ T-cells that produce IFNγ, we observed that this tendency is sustained among tumors that are colonized by CoPEC, reinforcing the possibility that this effect is confined to the regions that are populated with bacteria (Figure 6D). Accordingly, a negative correlation between IFNγ was observed in tumors that were subcutaneously implanted in wild-type mice that were colonized by CoPEC (Supplementary Figure S7). Furthermore, the expression of CD8, CD4 and CCR5 were significantly lowered within right-sided CRC tumors with a greater expression of LPCAT2 in pks^+^ tumors (Supplementary Figure S8A-D). By applying spatial profiling approaches, we were able to quantify the areas of overlap between CD8^+^ T-cells and glycerophospholipids. Interestingly, we observed a significant inverse correlation between the quantification of CD8^+^ spots and PC intensity (*r* = -0.56, *p* = 0.04) in the CoPEC patients (Figure 6E). Equally of importance, the expression of the genes encoding for CXCL5, CCL4 and CXCR1 that are involved in the recruitment of neutrophils was significantly lowered within right-sided CRC tumors with a high-glycerophospholipid microenvironment (Supplementary Figure S9A-D). By contrast, this correlation was lost in CoPEC-negative tumors (*r* = -0.04, *p* = 0.81) (Figure 6F), suggesting CD8^+^ T-cell expression is tightly intertwined with a specific metabolic pathway in a Colibactin-induced immune-suppressive microenvironment in right-sided CRC.

## Discussion

It has been established that patients with right-sided CRC exhibit a worse prognosis and differences in their tumor-associated biofilms^2,18^ and lipid metabolism when compared to left-sided CRC.^22^ However, it remains unclear whether the tumor-associated microbiota may influence the metabolic reprogramming of the tumor microenvironment and how can lead to variable responses to chemotherapies. To our knowledge, this is the first study showing that CoPEC can induce tumor heterogeneity in lipid metabolism, which in turn facilitates the progression of colon cancer and chemoresistance.

Herein we identified that the genus *Escherichia shigella* was the predominant bacterial population in tumors from relapsing patients with right-sided CRC cancer. A majority of detected bacteria in pks^+^ tumors were previously associated with the development of intestinal inflammation, CRC, or other inflammatory bowel disease. For instance, *Bacteroides fragilis* has *B. fragilis* toxin (bft) gene that produces fragilysin or BFT. BFT disrupts tight contacts, and increases the permeability and damage of the intestine, thus being involved in the development of inflammation and carcinogenic processes.^23,24^ Another *Bacteroides* species B. *uniformis* and *B. stercoris* previously were found to be significantly enriched in participants with bloody stools and were associated with higher risk to develop CRC.^25^ *Lachnoclostridium* species have been presented as promising bacterial markers for the noninvasive stool-based diagnosis of CRC adenoma.^26^ *Barnesiella* species were found to be associated with systemic inflammation.^27^ *Odoribacter* species in the previous studies have been correlated with somatic mutations and cell proliferation in CRC patients alongside decreased abundance in cystic fibrosis, nonalcoholic fatty liver disease, and inflammatory bowel disease.^28–30,31^ Conducting a more thorough analysis with a larger cohort of patients with left-sided CRC in the future is essential to draw meaningful conclusions.

Previous works have shown that CoPEC induces double-strand DNA breaks, DNA mutations and can modulate the tumor microenvironment to favor the emergence of senescent cells, affecting tumor progression.^8,12,19^ Herein, it is clear that CoPEC infection increases oxidative stress in colon cancer cells, which may lead to the formation of end-products of ROS-mediated lipid peroxidation. This observation is consistent with the macrocyclic Colibactin activity, which induces DNA double-strand breaks via copper-mediated oxidative cleavage.^32^ However, it remains a prerequisite to defining how differences between cancer cells can lead to variable responses to chemotherapies and subsequently tumor relapse. However, bulk analysis obscured our understanding of how the efficacy of chemotherapy is locally modulated by intratumoral bacteria because this approach cannot provide insights into the molecular events that locally occurred either sequentially or in parallel throughout the proliferation of resistant tumor cells.^5,33^ To address this issue, we applied state-of-the-art high-throughput *in situ* spatial profiling technologies to define whether CoPEC may locally contribute to metabolic changes that result in non-responsiveness to chemotherapeutic challenges and may subsequently create a potential vulnerability to tumor regrowth. This led us to identify that the intra-tumoral areas that are populated by Colibactin-producing bacteria contribute to unbalanced lipid metabolism that may provide the breeding ground for the emergence of resistant cancer cells. Specifically, we identified overexpression of key enzymes of lipid metabolism, that may account for the observed differences in PA, PC, PI, PE, PS, and PG intensity in bacteria microniches between pks^+^ and -pks^−^ groups, particularly correlated with PC, PA, and ceramide metabolism. Notably, we observed an increase in DGKG levels in tumors colonized by CoPEC when comparing transcripts from the relapse group. Several members of the DGK family have been implicated in CRC.^34^ In particular, DGKG plays a role in DNA methylation in CRC tumors, suggesting it is an early event during CRC tumorigenesis.^34^ In line with altered PC metabolism in CRC,^35^ we also identified overexpression of LPCAT2, which is part of the Lands cycle and is involved in the reacylation of LPC into PC. Of note, PC and PI are generally increased in CRC and have been associated with cancer development and progression.^36,37^ Furthermore, PS can be synthesized from PC and PE and plays an important role in mitochondrial function and apoptosis.^38^ LPCAT2 has been associated as an important enzyme in inflammatory cells and is up-regulated by LPS stimulation.^39^ Furthermore, we noticed a change in the expression of enzymes from the cytosolic phospholipase A2 (cPLA2) family that is also involved in the reacylation of the Lands cycle. On the one hand, PLA2G16, PLA2G4D and PLA2G2F were up-regulated in pks^+^ tumors. This result is in agreement with previously published data where cPLA2α is elevated in senescent T cells in the tumor microenvironment.^40^ On the other hand, PLA2G6 (cPLA2 group VI) gene which encodes for a calcium-independent PLA2, was down-regulated in the tumors colonized by CoPEC. It was accompanied by overexpression of genes mRNAs involved in the decrease of ceramide (e.g., ACER2 and SGMS1). This accords with the lowered generation of ceramide upon inhibition of cPLA2 that enhances colon tumorigenesis^41^ and with the accumulation of glycerophospholipids that play an important role in the autophagy pathway.^42^

Lipid droplets serve as an energy reserve for cancer cells that require a substantial amount of energy for their rapid growth and proliferation. The CoPEC-induced dysregulation in lipid metabolism may affect several cell functions. Notably, lipid droplet accumulation may modulate signaling pathways that contribute to cell proliferation and chemoresistance.^43,44^ Of note, a recent work described that *Fusobacterium nucleatum* promotes CRC cancer cells to acquire stem cell‐like features by lipid droplet‐mediated Numb degradation.^45^ Among different functions, lipid droplets can *i*) protect membranes from peroxidation reactions under oxidative stress conditions and maintain organelle homeostasis; *ii*) regulate autophagy by different mechanisms; *iii*) respond to exogenous lipid overload to reduce the accumulation of lipotoxic lipids and others; and iv) may promote acidosis-driven epithelial-to-mesenchymal transition. Accordingly, lipogenesis is heightened in cancer cells that were infected by CoPEC but not by mutant bacteria that do not produce colibactin. In our study, Spider Mass technology revealed that PC is a main structural component of lipid droplets in CoPEC HCT116 cells. Specifically, chemoresistant CRC cells upon 5-fluorouracil and oxaliplatin treatments *in vivo* and *in vitro*, showed lipid droplet accumulation and sensitivity is restored upon loss of LPCAT2, supporting PC synthesis.^21^ Interestingly, LPCATs enzymes participate, at different levels, in the regulation of lipid droplet formation, which plays a key role in lipid droplet membrane synthesis and expansion through PC biosynthesis.^46^ In addition, LPC and ceramides were down-regulated in the same conditions. Ceramide is a central molecule of sphingolipid metabolism and is important to maintain cell homeostasis through an orchestrated balance between cell proliferation and apoptosis.^47^ Studies suggest that cancer cells may acquire the ability to maintain ceramide low levels, bypassing normal metabolic control and then, contributing to chemoresistance.^48,49^ These data suggest lipid metabolic reprogramming of tumor cells as a possible alternative route supporting triacylglycerol, potentially through modulation of PC by cPLA2 of the Lands cycle. Together, these data suggest that oxidative stress potentially induces a cascade of events in the DNA damage and can trigger lipid droplet accumulation, supporting chemoresistance.

It is important to highlight that chemotherapy efficacy is in part a result of its ability to enhance adaptive antitumor immune responses.^50^ Our analysis provided evidence of a lowered infiltration of IFNγ-producing CD8^+^ T-cells in microniches that are populated by bacteria, mainly in patients colonized by CoPEC. To support this finding, we examined how CoPEC infection affected tumor growth using MC38 subcutaneous tumor model *in vivo* model. Similarly, tumor volume was negatively correlated with IFNγ, corroborating with data in the right-sided CRC tissues from patients colonized by CoPEC. In support, Lopès et al.^51^ showed a decrease of CD3^+^ and CD8^+^ T-cells correlated with the anti-PD-1 immunotherapy efficacy in mice infected by CoPEC. Likewise, a work focused on the spatial effect of the intratumoral microbiota in cancer, reported an increase in CD11b^+^ and CD66b^+^ myeloid cells but lowered densities of CD4^+^ and CD8^+^ T-cells in bacteria-positive microniches when compared to bacteria-negative areas.^5^ Further work is now needed to determine whether this lowered intratumoral accumulation of IFNγ-producing CD8^+^ T-cells may result from a decreased immunogenicity of tumor cells. In support of this possibility, it has been established that LPCAT2 expression modulates the presence of calreticulin at the surface of tumor cells.^21^ Alternatively, one may postulate that there is a decreased motility of IFNγ-producing CD8^+^ T-cells within infected tumors as what observed in response to Echovirus 30 infection.^52^

Therefore, our results using spatially resolved metabolomic and transcriptomic approaches clarify how the presence of Colibactin-producing bacteria may locally establish tumor heterogeneity for evading immune surveillance. These findings provide unique insights for development of novel therapeutic intervention for mitigating resistance to chemotherapies in right-sided CRC.

## Materials and methods

### Patient samples

All patients included in the analysis were diagnosed as sporadic cases and with tumors that arise in the right- or left-sided CRC. We used 101 samples of the Biobanks that have been set up at the Hospital Henri Mondor (*n* = 44) and the Hospital of Clermont-Ferrand (*n* = 57). A part of the patients were enrolled in several prospective cohorts named CCR1–3 (Acronyms Valihybritest and Vatnimad; for description, see Sobhani et al.^53^ and on ClinicalTrials.gov: NCT01270360). This protocol has been approved by the ethics committee of *Comité de Protection des Personnes Paris Est-Henri Mondor* (no. 10–006 in 2010). Another part of these patients underwent surgery for CRC in the Digestive and Hepatobiliary Surgery Department of the University Hospital of Clermont – Ferrand. All patients were adult volunteers and signed informed consent before they were included in the study.

### Identification of pks^+^ and pks^−^ tumors from right-sided CRC patients

We used two methods to validate the presence of *pks* island in the right-sided CRC tumor tissue. First, for patients from Hospital of Clermont-Ferrand and Créteil, the samples were analyzed by PCR using specific primers located in the *clbB* and *clbN* genes of the *pks* island: *clbBr* (r for reverse orientation) (5′-CCA TTT CCC GTT TGA GCA CAC-3′), *clbBf* (f for forward orientation) (5′-GAT TTG GAT ACT GGC GAT AAC CG-3′), *clbNr* (5′-CAG TTC GGG TAT GTG TGG AAG G-3′), and *clbNf* (5′-GTT TTG CTC GCC AGA TAG TCA TTC-3′).^54^

Posteriorly, for 65 patients from this study, DNA extraction was performed from eight 50 μm cryosections of nitrogen frozen tissue using the QIAamp PowerFecal DNA Kit® (Qiagen 12,830–50) following the manufacturer’s instructions with the following modification: 0.1 mm diameter silica beads were added to the lysis solution provided and shaking was performed at maximum speed for 10 minutes in a vibratory shaker. DNA concentration was measured using the Qubit dsDNA Broad Range assay kit (Invitrogen Q32853). qPCR reactions were performed on a QuantStudioTM 7 Flex Real-Time PCR System (Applied Biosystems 4,485,701) in 384-well plates with 32ng of DNA, in a final volume of 8 µl. Three technical replicates were performed for each sample. Primers and probes listed in the Table were used at a final concentration of 250 nM.

**Table:** 

Target	Primers	Reference
All bacteria 16S rDNA	F: 5’-CGGTGAATACGTTCCCGG-3’ R: 5’-TACGGCTACCTTGTTACGACTT-3’ Probe: 5’ FAM-CTTGTACACACCGCCCGTC-TAM 3’	*Suzuki et al*.^55^
*clbB*	F: 5’-GCGCATCCTCAAGAGTAAATA-3’ R: 5’-GCGCTCTATGCTCATCAACC-3’ Probe: 5’ FAM-TATTCGACACAGAACAACGCCGGT-TAM 3’	*Dejea et al*.^56^

The Master Mix Taqman (Applied Biosystems 4,440,038) was used with the following amplification steps: 50°C during 2 min, 95°C during 15 sec with 48 cycles, 60°C during 1 min.

The amount of *clbB* was determined using the QuantStudioTM Real-Time PCR software (version 1.7.2, Applied Biosystems) and the 2-ΔCt method, with all bacteria 16S rDNA as a calibrator. Patients with amplification were assigned as positive CoPEC.

### Microbiota analysis

A total of DNA from 76 right-sided CRC tumors and 11 left-sided CRC tumors tissues were used to perform microbiome analysis. Bacterial 16S rRNA gene V3-V4 region was amplified and sequenced on a MiSeq (2 × 250 bp; Illumina, Hayward, CA). Bioinformatic processing and statistical analysis were performed in R software environment as described previously.^57^ Briefly, paired-end fastq files without barcodes and adapters have been quality checked, denoised and prepared for further analysis using the dada2 package.^58^ Bacterial sequencies from amplicon sequence variant (ASV) table were annotated using latest Silva database (version 138.1).^59^ Data normalization and beta-diversity analysis were performed on each taxonomical level using DESeq2 package.^60^ To improve the power of detecting differentially abundant taxa, only those bacteria that appeared in 25% of samples at least in one of the compared groups were used for differential abundance analysis. Differences were considered significant when the corrected p-value (p adjusted) was < 0.05. Principal Coordinate Analysis (PCoA) based on Bray-Curtis distance matrix was performed to visualize complex data and to get a set of principal coordinates. PERMANOVA analysis (9999 permutations) was used to evaluate global differences between groups at all taxonomical levels.

### Tissue processing and FISH

Samples of right-sided CRC tumor tissue were collected within 30 minutes after surgical resection and immediately frozen in liquid nitrogen and stored at -80°C until further use. This analysis was conducted on 5 μm sections of 12 sporadic CRC patients’ samples using cryostat at -21°C. Serial adjacent sections were obtained for FISH and hematoxylin and eosin (H&E) staining. Tissues were visualized by a pathologist to obtain tumor area and bacteria were stained by FISH using the general bacterial rRNA probe, EUB338 conjugated with Alexa 555 as described previously.^61^

### Spatial metabolomic using MALDI-FTICR imaging

This analysis was conducted on 10 μm sections of the 77 right-sided CRC and 9 left-sided CRC patients obtained in a cryostat at -21°C and mounted on ITO slides. On each slide, a quality control section (rat kidney homogenate spiked with Rutine) was added. Imaging of the tumors was performed at 80 μm using a 7T-MALDI-FTICR in full scan mode (75–1000) and in negative ion mode. Following the acquisition, the MALDI matrix was removed in a bath of methanol and an H&E staining was performed. After, we conducted an untargeted metabolomics analysis between tumor tissue and tumor-adjacent stroma in two groups: relapse (24 tumors versus 21 tumors-adjacent stroma) and non-relapse (53 tumors versus 51 tumors-adjacent stroma). The normalized root-mean-square was applied and differential metabolite expression was determined using the limma package (v3.46.0) in R software. The following criteria were applied: p-value produced by Mann-Whitney-Wilcoxon test after FDR correction < 0.0005, fold change > 2 or < -2). In a subgroup of patients, we performed an *in situ* metabolites analysis dependent on the presence of bacteria in 12 sporadic right-sided CRC patients, who were previously grouped as pks^+^ and -pks^−^ patients. In addition, quantification was also performed in the total tissue of 65 right-sided CRC patients in relation to pks status, acquiring the metabolite intensity of all tumor tissue. The normalized root-mean-square was applied and differential metabolite expression was determined using the limma package (v3.46.0) in R software. The following criteria were applied: p-value produced by Mann-Whitney-Wilcoxon test after FDR correction < 0.05, fold change > 1 or < -1). Volcano plot was used to present differential expressed metabolites with the EnhancedVolcano R package (version 4.0.3). Data acquisition, processing, and data visualization were performed using FlexImaging 4.1 and Multimaging 1.1 (ImaBiotech SAS, France). MSI data were acquired from each tissue section as well as matrix control areas adjacent to the tissue sections to check for analyte dispersion during sample preparation. Metabolite annotation was performed according to Lipid maps (http://www.lipidmaps.org/) and Metlin (https://metlin.scripps.edu).

### RNA sequencing (RNAseq) and CMSclassifier from right-sided CRC tumors

Total RNA was extracted from 47 right-sided CRC and 10 left-sided CRC tumor samples using TRIzol®-chloroform extraction method. RNA was sent to the NOVOgene company (China) that carried out the quality control, library preparation, and sequencing. The reads quality was assessed using FastQC (v0.11.4)^62^ combined with MultiQC (v1.6).^63^ We performed splice-aware alignment on RNA-seq using the STAR transcriptome aligner (v2.5.0)^64^ with human genome version GRCh38 from Ensembl release 99.^65^ After alignment, featureCounts (subread v 1.6.1) was used to obtain the matrix of counts of fragments by genes from sorted BAM files.^66^ We removed samples for which less than 50% of the fragments mapped to genes and used blast+ (2.9.0) to align the reads on the nt collection and confirm that the lack of alignment to human genes was due to an excess of reads mapping to non-human organisms, suggesting contamination. We filtered gene-level raw counts by removing all genes with no fragment mapped in more than half of the samples. CMS classifications were determined using the CMScaller R package.^67,68^ We used the recommended option ‘RNA-seq=TRUE’, which makes CMScaller perform a log2 transformation and quantile normalization and the option ‘FDR = 0.05’. Normalized counts for further use were generated by applying a quantile normalization using the voom function of the limma package (v3.46.0)^69^ and Volcano plot was used to present differential expressed genes with the EnhancedVolcano R package (version 4.0.3).

### Whole exome sequencing (WES) analysis

Sequencing data were aligned to human genome GRCh38 with BWA v0.7.17.^70^ Aligning sequence reads, clone sequences and assembly contigs with BWA-MEM. arXiv:1303.3997v1 [q-bio.GN].]. Duplicate reads were marked with sambamba v0.6.8.^71^ GATK v4.1.9 was used to perform based recalibration using dbSNP v152^72^ and Ryan Mills et al.^73^ as reference list of known variable sites. Somatic variants were called using Mutect2^74^ integrated in GATK v4.1.9. Finally, the variants were annotated using snpEff v5.0c.^75^ The genetic impact on the CoPEC-induced metabolic adaptation of cancer cells was evaluated by whole exome sequencing of 21 right- and 10 left-sided CRC tumors and their adjacent normal tissue.

### Cell culture, bacterial infection, lipid droplets, and ROS

Human HCT116 cells or MC38 cell line, derived from methylcholanthrene-induced C57BL6 murine colon adenocarcinoma cells were used in this study. HCT116 cells were maintained in advanced DMEM F12 and MC38 were maintained in DMEM GlutaMAX. These cells were supplemented with 10% FBS, 1 mM L-Glutamine (except to MC38), and Penicillin (100 µ/ml) – Streptomycin (0.1 mg/ml), at 37°C under 5% CO2 pressure. Cells were routinely tested for mycoplasma. All media components were obtained from Sigma-Aldrich. Treatments were carried out using the clinical CoPEC 11G5 strain isolated from a patient with CRC and its isogenic mutant 11G5*ΔclbQ*, depleted for the *clbQ* gene in the *pks* island and unable to produce Colibactin.^24^ These strains were grown at 37°C in Luria-Bertani medium overnight. For bacterial infections, cells were infected at a multiplicity of infection (MOI) of 10 or 100 bacteria for 3 h and cells remained in culture for 1 h or 5 days after infection. Cells were washed three times with PBS and culture medium with 200 μg/mL gentamicin was added.^17^ Lipid droplets were analyzed using Nile red (Molecular Probes) at 1:2000 in PBS for 15 min and then fixed in 4% paraformaldehyde for 10 min at 4°C. The slides were mounted in ProLong Gold Antifade with DAPI (Molecular Probes) before imaging. Lipid droplets quantification was performed by counting red lipid bodies on merged pictures (300 cells per cell line). To measure cellular ROS, cells were stained using 5 μM CellRox Deep Red Reagent (Invitrogen, C10422) for 30 min at 37^◦^C, washed three times with PBS and fixed using 4% paraformaldehyde for 10 min before imaging. The slides were mounted in ProLong Gold Antifade with DAPI (Molecular Probes) before imaging. A semi-quantitative assessment of the percentage of ROS in 300 cells. The scores were as follows: score 0 (absence of CellRox fluorescence); score 1 (weak CellRox fluorescence); score 2 (moderate CellRox fluorescence) and score 3 (strong CellRox fluorescence). Data are expressed by the percentage of cells in each score and group (two independent experiments and duplicates for each experiment). Images were acquired with an Axio Imager M2 (Zeiss) coupled with an Apotome.2 (×63 or x100 objective).

### Spider mass analysis

After almost 70% confluence, HCT116 and MC38 cells were washed two times with DPBS, dried under PSM for 10 min at room temperature, and then analyzed by the SpiderMass directly into the cell plate. The overall layout of the SpiderMass setup has already been covered elsewhere.^76^ In brief, the system is made up of three parts: the mass spectrometer itself, a laser system for remote micro-sampling of tissues and a transfer line allowing for the transfer of the micro-sampled material. In this study, the laser intensity was set to 4 mJ/pulse and a 200 µL/min infusion of isopropanol was administered during each acquisition. 200 µg/mL of Leucine enkephalin was added to the infusion to play the role of a lockmass. The acquisition was composed of a burst of 10 laser shots resulting in an individual spectrum. Spectral acquisition was performed both in negative ion mode in sensitivity mode and the mass range was set to m/z 50–2000. The raw files were imported into “Abstract Model Builder” - AMX (version 1.0 1972.0, Waters, Hungary) to perform multivariate statistical analyses using linear discriminative analyses (LDA). Discriminative ions were found looking at each dual condition loading plot. Boxplots for each specific ions were obtained thanks to Kruskal-Wallis significant tests.

### RNAseq analysis from MC38 cell line

MC38 cells were infected using the 11G5 strain and 11G5*ΔclbQ* strain at a MOI of 100 bacteria during 3 h and cells remained in culture for 1 h after infection. Non-infected cells also were analyzed under the same conditions. Posteriorly, cells were immediately lysed in RLT buffer and RNA was extracted using RNeasy Mini Kit (QIAGEN) following manufacturer protocol. mRNA library preparation was realized following the manufacturer’s recommendations (Illumina Stranded mRNA Prep Kit from ILLUMINA). Final samples pooled library prep was sequenced on Novaseq 6000 ILLUMINA with S1-200cycles cartridge (2×1600Millions of 100 bases reads) corresponding to 2 × 30Millions of reads per sample after demultiplexing.

### Cell viability

Cell viability was investigated using WST-1 assay reagent (ab155902). This test is based on the cleavage of the tetrazolium salt WST-1 to formazan by cellular mitochondrial dehydrogenases. 5 × 10^3^ MC38 cells were infected with 11G5 strain and 11G5∆clbQ strain at MOI of 500 bacteria during 3 h and cells remained in culture for 7 days after infection. Non-infected cells were used as a negative control. All cells were treated with 0 and 20 μg/mL of oxaliplatin in 96-well plate at 37°C for 72 h. Posteriorly, 3 weeks post-infection with 11G5 strain, treatments were carried out for 1 week with 0 and 5 μg/mL of oxaliplatin (Accord Healthcare Limited) in the absence or presence of Triacsin C (10 µM, Cayman Chemical). WST-1 solution was added and incubated for 4 h at 37°C. Absorbance was measured using FLUOstar Omega plate reader (BMG Labtech) at 420 nm. The percentage of cell viability was calculated in relation to non-infected cells without oxaliplatin.

### Spatial transcriptomic using RNAscope insitu hybridization

RNAscope multiplex fluorescent reagent 2.5 HD kit assay (Advanced cell diagnostics, Newark, CA, USA) was performed using 10 frozen right-sided CRC tissue (5 samples for each pks^+^ and pks^−^ groups) and cryostat cut sections of 10 µm were collected and mounted onto Superfrost™ Plus microscope slides. Slides were immersed in the 4% PFA for 1 h at 4°C. Posteriorly, the sections were dehydrated and slides were baked for 1 h at 60°C and a hydrophobic barrier was drawn around the tumor tissue. Endogenous peroxidases were blocked using a hydrogen peroxide solution for 10 min at room temperature and then, was used RNAscope protease IV sob under the same conditions. The probes hybridization process was performed using the HybEZ™ II oven for 2 h at 40°C for Hs-IFNγ (310501, Opal 570) and Hs-CD8A (560391-C3, Opal 520) probes. Between each amplification and staining step, slides were washed twice in 1X RNAscope wash buffer for two minutes. Then, the slides were incubated with DAPI (ThermoFisher Scientific) for 3 min at room temperature and placed with ProLong Gold antifade mounting solution (ThermoFisher Scientific) prior to imaging. Programmed cell counting using Imaris (Bitplane version 9.5.0) to count the number of spots for CD8A (5000–7000 cells, approximately) and for CD8A and IFNγ in the same cell (500–1000 cells, approximately).

### Mice infection and tumor growth

Animal protocols were approved by the Ministère de l’Education Nationale, de l’Enseignement Supérieur et de la Recherche (APAFIS#20990). This study was performed using male wild-type C57BL/6J mice. All mice were housed in conventional conditions at the animal care facility of the Institute Pasteur of Lille and had unlimited access to food and water. The colonization by CoPEC using wild-type (WT) mice was conducted as described previously.^73^ In brief, to enhance *Escherichia coli* strain colonization, we administered streptomycin (2.5 g/l) for 3 days prior to oral inoculation with 11G5 strain (*n* = 7) or its isogenic mutant 11G5*∆clbQ* (*n* = 6) (≈1 × 10^9^ bacteria in PBS). Eight days after infection, to induce tumor formation, 5 × 10^5^ MC38 cells in PBS were injected subcutaneously into the left flank of male mice. Tumor volume in mm^3^ was monitored two or three times a week by the measurement of two perpendicular diameters using a caliper according to the formula L×S2/2, where L and S are the largest and smallest diameters in mm, respectively.

### RNA extraction and qRT-PCR from mice samples and cell

Tumors and colon tissues were homogenized with ceramic beads on a MagNA Lyser (Roche). RNA was extracted using RNeasy Mini Kit (QIAGEN) following manufacturer protocol. 250ng RNA of each sample was retro-transcribed using Affinity Script cDNA synthesis kit (Agilent Technologies). 5ng of cDNA was used for the qRT-PCR reaction using Brilliant III Ultrafast SYBR Green QPCR master mix (Agilent Technologies) on AriaMx qRT-PCR system (Agilent Technologies). The following primers were used: mIFN-γ For= GCTTTGCAGCTCTTCCTCAT and Rev= CCAGTTCCTCCAGATATCCAAG; mLpcat2 For=TCCCAGAAGGTACTTGTACTAATCG and Rev=TGTTTGGGTATCTGAGGAGGA.

### Statistical analysis

Overall survival was evaluated using the Kaplan – Meier method available in the R (version 4.0.3) package survival. The *p* values are from log-rank tests. Pearson correlation was used to investigate the correlation between PC and CD8^+^ T-cell spots with R software (version 4.0.3) and GraphPad Prism software (version 6.0). Statistical analyses between two groups were performed with the student’s t-test or a Mann-Whitney U test, conforming to the results of the normality test. A one-way ANOVA followed by a posttest Bonferroni correction also was used when appropriate, using GraphPad Prism software (version 6.0). A p-value less than 0.05 was considered statistically significant.

## Supplementary Material

Supplemental Material
