# Supplementary material for: The colibactin-producing Escherichia coli alters the tumor microenvironment to immunosuppressive lipid overload facilitating colorectal cancer progression and chemoresistance
Source: Gut Microbes. 2024 Feb 28;16(1):2320291. doi: 10.1080/19490976.2024.2320291 (PMC10903627; doi:10.1080/19490976.2024.2320291)
Supplement: Supplemental Material [file KGMI_A_2320291_SM6627.zip › Supplementary_Tables.docx]

**Table S1: Beta diversity between the relapse and non-relapse groups from right-sided colorectal cancer patients.**

| Row.names | baseMean | log2FC | lfcSE | stat | pvalue | padj | mean_relapse | mean_non_relapse | SD_relapse | SD_non_relapse |
| --- | --- | --- | --- | --- | --- | --- | --- | --- | --- | --- |
| Class_Fusobacteriia | 4130.87796 | -3.2160893 | 0.76498085 | -4.2041435 | 2.6207E-05 | 0.00011793 | 1.201 | 5.354 | 1.679 | 9.622 |
| Class_Gammaproteobacteria | 2663.83382 | 1.02561961 | 0.3673144 | 2.79221181 | 0.00523491 | 0.01570472 | 10.066 | 4.126 | 13.646 | 6.643 |
| Class_Negativicutes | 3093.87301 | -2.1081623 | 0.46146476 | -4.5684145 | 4.9143E-06 | 4.4228E-05 | 1.810 | 5.104 | 1.564 | 7.143 |
| Family_Enterobacteriaceae | 1592.80217 | 1.87258764 | 0.6536744 | 2.86471006 | 0.00417391 | 0.04464082 | 8.349 | 2.475 | 13.933 | 6.208 |
| Family_Erysipelatoclostridiaceae | 553.28994 | 0.96534017 | 0.34216147 | 2.8213 | 0.00478295 | 0.04464082 | 1.295 | 0.878 | 0.751 | 0.763 |
| Family_Fusobacteriaceae | 3784.98597 | -3.4360968 | 0.79509118 | -4.3216387 | 1.5487E-05 | 0.00043365 | 0.841 | 5.065 | 1.128 | 9.603 |
| Family_Marinifilaceae | 252.952532 | 1.53372707 | 0.5054081 | 3.03463095 | 0.0024083 | 0.03371622 | 0.564 | 0.582 | 0.704 | 1.806 |
| Family_Rhizobiaceae | 16.2053932 | -3.1910687 | 0.99297529 | -3.2136436 | 0.00131062 | 0.02446495 | 0.004 | 0.041 | 0.011 | 0.080 |
| Family_Selenomonadaceae | 2103.72438 | -4.9908931 | 1.05075166 | -4.7498313 | 2.0359E-06 | 0.00011401 | 0.191 | 3.047 | 0.418 | 6.933 |
| Genus_Anaerotruncus | 18.5719263 | -2.853638 | 0.96274535 | -2.9640631 | 0.00303606 | 0.04326385 | 0.020 | 0.150 | 0.041 | 0.785 |
| Genus_Bacteroides | 13534.701 | -0.5619464 | 0.187164 | -3.0024277 | 0.00267836 | 0.04326385 | 26.651 | 28.248 | 15.646 | 14.366 |
| Genus_Centipeda | 83.676953 | -9.2071021 | 2.03399523 | -4.5266095 | 5.9938E-06 | 0.00068329 | 0.008 | 0.740 | 0.026 | 2.807 |
| Genus_Escherichia-Shigella | 1518.66648 | 2.39561095 | 0.67791663 | 3.53378402 | 0.00040966 | 0.01167519 | 8.729 | 2.587 | 15.067 | 6.506 |
| Genus_Fusobacterium | 2414.21728 | -2.6994167 | 0.75435877 | -3.5784256 | 0.00034567 | 0.01167519 | 0.882 | 5.310 | 1.161 | 10.152 |
| Genus_Lachnoclostridium | 815.282249 | 0.92584133 | 0.21514148 | 4.3034069 | 1.6819E-05 | 0.00095869 | 3.415 | 1.458 | 4.344 | 1.616 |
| Genus_Lactococcus | 6.17881642 | -4.6714996 | 1.3712645 | -3.4067094 | 0.00065751 | 0.01499125 | 0.000 | 0.027 | 0.001 | 0.082 |
| Genus_Selenomonas | 963.106567 | -3.2890765 | 1.09153167 | -3.0132671 | 0.00258451 | 0.04326385 | 0.195 | 2.306 | 0.449 | 5.405 |
| Order_Enterobacterales | 2351.85979 | 2.33519422 | 0.60136153 | 3.8831786 | 0.0001031 | 0.00159805 | 8.604 | 2.701 | 13.980 | 6.208 |
| Order_Fusobacteriales | 3730.29509 | -2.6820916 | 0.77621813 | -3.4553323 | 0.00054961 | 0.00567935 | 1.202 | 5.354 | 1.681 | 9.622 |
| Order_Veillonellales-Selenomonadales | 2806.63453 | -2.6842664 | 0.66811336 | -4.0176811 | 5.8774E-05 | 0.00159805 | 1.045 | 4.327 | 1.173 | 7.369 |
| Phylum_Fusobacteriota | 3198.50249 | -2.8768949 | 0.75077253 | -3.8319128 | 0.00012715 | 0.00063575 | 1.195 | 5.324 | 1.675 | 9.561 |
| Phylum_Proteobacteria | 4422.10686 | 0.83368347 | 0.32681591 | 2.55092682 | 0.01074369 | 0.02685922 | 12.215 | 7.045 | 13.941 | 7.776 |
| Species_Bacteroides plebeius | 541.134045 | -7.2279654 | 1.73215211 | -4.1728237 | 3.0085E-05 | 0.00210593 | 0.491 | 1.895 | 1.545 | 4.892 |
| Species_Colidextribacter massiliensis | 7.01779314 | -5.0833997 | 1.43831303 | -3.5342792 | 0.00040889 | 0.00954074 | 0.011 | 0.051 | 0.049 | 0.143 |
| Species_Dialister pneumosintes | 124.653708 | -3.1625328 | 1.0758905 | -2.939456 | 0.00328789 | 0.03835871 | 0.144 | 0.460 | 0.366 | 1.062 |
| Species_Escherichia-Shigella coli | 1178.90176 | 2.69178989 | 0.71226402 | 3.77920238 | 0.00015733 | 0.0055066 | 10.087 | 2.637 | 18.053 | 6.038 |
| Species_Fusobacterium nucleatum | 1311.91639 | -2.8817229 | 0.89928838 | -3.2044481 | 0.00135322 | 0.0236813 | 0.993 | 4.152 | 2.154 | 8.617 |
| Species_Lactococcus lactis | 4.62062316 | -4.1638476 | 1.37458909 | -3.0291581 | 0.00245236 | 0.03433309 | 0.001 | 0.045 | 0.002 | 0.147 |

**Table S2. Beta-diversity analysis in relapse patients’ group**

|  | **Bacteria** | **baseMean** | **log2FoldChange** | **pvalue** | **padj** | **Taxonomy group** | **Mean value in right-sided colon samples** | **Mean value in left-sided colon samples** |
| --- | --- | --- | --- | --- | --- | --- | --- | --- |
| 1 | Family_Barnesiellaceae | 119.7 | 9.2 | 0.00054 | 0.01926 | Family | 0.33 | 0.00 |
| 2 | Family_Leptotrichiaceae | 43.6 | 21.2 | 0.00000 | 0.00000 | Family | 0.35 | 0.00 |
| 3 | Genus_[Ruminococcus] gauvreauii group | 54.1 | 21.6 | 0.00000 | 0.00000 | Genus | 0.13 | 0.00 |
| 4 | Genus_Alloprevotella | 19.0 | 20.2 | 0.00001 | 0.00042 | Genus | 0.22 | 0.00 |
| 5 | Genus_Barnesiella | 50.1 | 20.2 | 0.00000 | 0.00000 | Genus | 0.29 | 0.00 |
| 6 | Genus_Lachnospiraceae UCG-003 | 10.5 | 19.4 | 0.00002 | 0.00075 | Genus | 0.08 | 0.00 |
| 7 | Genus_Leptotrichia | 37.2 | 19.9 | 0.00000 | 0.00000 | Genus | 0.37 | 0.00 |
| 8 | Phylum_Bacteroidota | 24919.3 | -1.7 | 0.00218 | 0.01525 | Phylum | 35.99 | 52.52 |
| 9 | Species_[Ruminococcus] torques group lactaris | 68.0 | 21.7 | 0.00000 | 0.00000 | Species | 0.36 | 0.00 |
| 10 | Species_Alistipes inops | 3.5 | -24.5 | 0.00000 | 0.00000 | Species | 0.05 | 0.03 |
| 11 | Species_Clostridium sensu stricto 1 perfringens | 39.7 | 20.9 | 0.00000 | 0.00000 | Species | 0.41 | 0.00 |
| 12 | Species_Enterococcus faecalis | 1359.9 | -11.0 | 0.00283 | 0.04345 | Species | 0.14 | 6.28 |
| 13 | Species_Phascolarctobacterium faecium | 29.7 | 19.9 | 0.00000 | 0.00004 | Species | 0.43 | 0.00 |
| 14 | Species_Roseburia intestinalis | 33.8 | 20.7 | 0.00001 | 0.00011 | Species | 0.95 | 0.00 |
| 15 | Species_Ruminococcus bromii | 227.6 | 21.6 | 0.00000 | 0.00000 | Species | 0.92 | 0.00 |
| 16 | Species_Streptococcus gallolyticus | 100.0 | 22.0 | 0.00000 | 0.00000 | Species | 1.06 | 0.00 |

**Table S3. Beta-diversity analysis in non-relapse patients’ group**

|  | **Bacteria** | **baseMean** | **log2FoldChange** | **pvalue** | **padj** | **Taxonomy group** | **Mean value in right-sided colon samples** | **Mean value in left-sided colon samples** |
| --- | --- | --- | --- | --- | --- | --- | --- | --- |
| 1 | Class_Clostridia | 21892.7 | 1.5 | 4.0E-05 | 5.3E-04 | Class | 42.06 | 22.95 |
| 2 | Class_Lentisphaeria | 3.5 | 4.6 | 6.1E-03 | 2.6E-02 | Class | 0.01 | 0.00 |
| 3 | Class_Negativicutes | 2223.5 | 2.5 | 1.7E-04 | 1.1E-03 | Class | 4.12 | 1.04 |
| 4 | Family_Bacillaceae | 273.0 | -4.6 | 3.4E-04 | 4.5E-03 | Family | 0.16 | 1.41 |
| 5 | Family_Barnesiellaceae | 255.0 | 5.3 | 4.7E-06 | 7.6E-05 | Family | 0.62 | 0.09 |
| 6 | Family_Carnobacteriaceae | 279.8 | -5.7 | 2.5E-03 | 1.8E-02 | Family | 0.08 | 1.26 |
| 7 | Family_Caulobacteraceae | 112.9 | -3.3 | 4.9E-04 | 5.6E-03 | Family | 0.11 | 0.45 |
| 8 | Family_Clostridiaceae | 386.6 | 3.7 | 3.1E-03 | 2.1E-02 | Family | 0.86 | 0.05 |
| 9 | Family_Comamonadaceae | 188.1 | -3.3 | 4.7E-03 | 2.9E-02 | Family | 0.19 | 0.92 |
| 10 | Family_Enterobacteriaceae | 4173.1 | -5.0 | 2.2E-06 | 5.8E-05 | Family | 2.46 | 14.44 |
| 11 | Family_Leptotrichiaceae | 48.1 | 22.8 | 1.2E-21 | 5.0E-20 | Family | 0.30 | 0.00 |
| 12 | Family_Mitochondria | 1616.0 | -3.3 | 4.2E-06 | 7.6E-05 | Family | 1.71 | 5.67 |
| 13 | Family_Rhizobiaceae | 60.9 | -4.1 | 1.9E-03 | 1.5E-02 | Family | 0.04 | 0.32 |
| 14 | Family_Ruminococcaceae | 4617.6 | 1.4 | 1.4E-03 | 1.4E-02 | Family | 11.89 | 3.49 |
| 15 | Family_Selenomonadaceae | 1317.6 | 27.5 | 1.7E-46 | 1.4E-44 | Family | 2.13 | 0.00 |
| 16 | Family_Streptococcaceae | 1477.5 | -2.3 | 6.5E-03 | 3.7E-02 | Family | 1.73 | 2.50 |
| 17 | Family_Unknown Family | 19.4 | -3.7 | 1.7E-03 | 1.5E-02 | Family | 0.02 | 0.08 |
| 18 | Genus_[Eubacterium] eligens group | 195.8 | 4.0 | 6.1E-03 | 4.3E-02 | Genus | 0.30 | 0.01 |
| 19 | Genus_[Eubacterium] hallii group | 237.3 | 2.6 | 1.1E-03 | 1.1E-02 | Genus | 0.50 | 0.14 |
| 20 | Genus_[Eubacterium] siraeum group | 79.6 | 5.9 | 3.4E-03 | 2.6E-02 | Genus | 0.16 | 0.00 |
| 21 | Genus_[Eubacterium] ventriosum group | 32.7 | 5.3 | 1.5E-05 | 3.0E-04 | Genus | 0.06 | 0.00 |
| 22 | Genus_Acidibacter | 16.6 | -3.2 | 6.6E-03 | 4.4E-02 | Genus | 0.02 | 0.09 |
| 23 | Genus_Agathobacter | 581.5 | 5.0 | 1.7E-04 | 2.1E-03 | Genus | 1.24 | 0.18 |
| 24 | Genus_Anaerobacillus | 223.4 | -3.9 | 3.7E-03 | 2.8E-02 | Genus | 0.15 | 1.54 |
| 25 | Genus_Anaerostipes | 311.5 | 4.5 | 6.1E-06 | 1.4E-04 | Genus | 0.51 | 0.06 |
| 26 | Genus_Blautia | 1665.0 | 2.0 | 1.2E-03 | 1.2E-02 | Genus | 3.25 | 1.45 |
| 27 | Genus_Butyrivibrio | 23.3 | 22.2 | 3.6E-07 | 1.1E-05 | Genus | 0.48 | 0.00 |
| 28 | Genus_Clostridium sensu stricto 1 | 437.6 | 4.4 | 6.5E-04 | 7.2E-03 | Genus | 0.91 | 0.04 |
| 29 | Genus_Coprobacter | 24.9 | 6.5 | 1.1E-04 | 1.6E-03 | Genus | 0.06 | 0.00 |
| 30 | Genus_Erysipelotrichaceae UCG-003 | 301.7 | 4.3 | 6.9E-05 | 1.1E-03 | Genus | 0.61 | 0.05 |
| 31 | Genus_Escherichia-Shigella | 3228.8 | -3.6 | 1.9E-03 | 1.7E-02 | Genus | 2.57 | 15.66 |
| 32 | Genus_Faecalibacterium | 4875.0 | 3.1 | 3.0E-05 | 5.1E-04 | Genus | 8.45 | 1.40 |
| 33 | Genus_Granulicatella | 170.3 | -5.1 | 7.0E-03 | 4.5E-02 | Genus | 0.08 | 1.34 |
| 34 | Genus_Lachnospira | 204.5 | 6.0 | 3.4E-04 | 3.9E-03 | Genus | 0.33 | 0.01 |
| 35 | Genus_Lachnospiraceae ND3007 group | 313.5 | 5.2 | 3.2E-06 | 8.6E-05 | Genus | 0.50 | 0.02 |
| 36 | Genus_Lachnospiraceae NK4A136 group | 764.4 | 6.8 | 5.6E-14 | 3.5E-12 | Genus | 1.39 | 0.02 |
| 37 | Genus_Leptotrichia | 57.2 | 23.5 | 4.4E-23 | 4.2E-21 | Genus | 0.31 | 0.00 |
| 38 | Genus_Monoglobus | 66.4 | 4.7 | 1.2E-04 | 1.7E-03 | Genus | 0.10 | 0.01 |
| 39 | Genus_Oribacterium | 23.8 | 21.4 | 3.1E-13 | 1.5E-11 | Genus | 0.12 | 0.00 |
| 40 | Genus_Prevotella_7 | 436.6 | 26.2 | 1.6E-12 | 5.9E-11 | Genus | 0.90 | 0.00 |
| 41 | Genus_Prevotella_9 | 485.9 | 8.9 | 1.5E-03 | 1.5E-02 | Genus | 2.58 | 0.12 |
| 42 | Genus_Romboutsia | 73.2 | 5.2 | 2.5E-03 | 2.0E-02 | Genus | 0.24 | 0.01 |
| 43 | Genus_Roseburia | 1248.6 | 2.4 | 7.4E-03 | 4.7E-02 | Genus | 3.43 | 0.57 |
| 44 | Genus_Selenomonas | 862.5 | 25.6 | 1.4E-35 | 2.7E-33 | Genus | 2.09 | 0.00 |
| 45 | Genus_Slackia | 31.5 | 6.8 | 2.3E-03 | 1.9E-02 | Genus | 0.06 | 0.00 |
| 46 | Genus_Terrisporobacter | 73.0 | 7.2 | 4.2E-03 | 3.0E-02 | Genus | 0.09 | 0.01 |
| 47 | Genus_UCG-003 | 75.4 | 5.1 | 1.6E-05 | 3.0E-04 | Genus | 0.13 | 0.01 |
| 48 | Order_Bacillales | 213.7 | -4.0 | 1.8E-03 | 1.5E-02 | Order | 0.16 | 1.41 |
| 49 | Order_Caulobacterales | 98.6 | -2.9 | 2.1E-03 | 1.5E-02 | Order | 0.11 | 0.45 |
| 50 | Order_Clostridiales | 331.1 | 3.5 | 5.1E-03 | 2.5E-02 | Order | 0.86 | 0.05 |
| 51 | Order_Enterobacterales | 3708.9 | -3.5 | 5.7E-04 | 6.3E-03 | Order | 2.65 | 14.44 |
| 52 | Order_Gammaproteobacteria Incertae Sedis | 16.0 | -3.4 | 3.6E-03 | 2.1E-02 | Order | 0.02 | 0.08 |
| 53 | Order_Lactobacillales | 1499.2 | -2.2 | 6.0E-03 | 2.6E-02 | Order | 1.88 | 4.38 |
| 54 | Order_Monoglobales | 44.0 | 4.2 | 2.2E-04 | 3.3E-03 | Order | 0.09 | 0.01 |
| 55 | Order_Oscillospirales | 6611.3 | 1.1 | 6.6E-03 | 2.6E-02 | Order | 15.20 | 4.98 |
| 56 | Order_Rhizobiales | 83.7 | -3.1 | 3.8E-03 | 2.1E-02 | Order | 0.09 | 0.42 |
| 57 | Order_Rhodospirillales | 186.4 | 6.6 | 1.6E-04 | 3.3E-03 | Order | 0.46 | 0.00 |
| 58 | Order_Rickettsiales | 1363.0 | -2.8 | 1.2E-04 | 3.3E-03 | Order | 1.71 | 5.69 |
| 59 | Order_Staphylococcales | 349.1 | -3.9 | 7.4E-03 | 2.7E-02 | Order | 0.31 | 2.86 |
| 60 | Phylum_Firmicutes | 26585.8 | 0.8 | 3.7E-03 | 1.3E-02 | Phylum | 49.92 | 33.11 |
| 61 | Phylum_Proteobacteria | 5036.7 | -2.4 | 5.3E-06 | 3.7E-05 | Phylum | 7.06 | 25.07 |
| 62 | Species_[Ruminococcus] torques group lactaris | 146.7 | 24.3 | 1.5E-35 | 1.1E-33 | Species | 0.51 | 0.00 |
| 63 | Species_Alistipes inops | 11.4 | 20.0 | 7.8E-10 | 1.4E-08 | Species | 0.08 | 0.01 |
| 64 | Species_Alloprevotella tannerae | 22.7 | 22.2 | 2.3E-09 | 3.7E-08 | Species | 0.16 | 0.03 |
| 65 | Species_Anaerostipes hadrus | 218.1 | 3.9 | 3.5E-05 | 3.9E-04 | Species | 0.89 | 0.08 |
| 66 | Species_Bacteroides eggerthii | 248.7 | 8.4 | 1.6E-03 | 1.3E-02 | Species | 0.75 | 0.07 |
| 67 | Species_Bacteroides nordii | 55.8 | 6.4 | 2.6E-03 | 1.8E-02 | Species | 0.22 | 0.18 |
| 68 | Species_Blautia obeum | 451.6 | 2.7 | 3.4E-04 | 3.3E-03 | Species | 1.72 | 0.30 |
| 69 | Species_Butyrivibrio crossotus | 29.0 | 22.6 | 1.1E-07 | 1.6E-06 | Species | 0.94 | 0.00 |
| 70 | Species_Clostridium sensu stricto 1 paraputrificum | 21.0 | 21.2 | 1.8E-12 | 4.4E-11 | Species | 0.08 | 0.00 |
| 71 | Species_Clostridium sensu stricto 1 perfringens | 37.9 | 22.9 | 1.0E-12 | 3.0E-11 | Species | 0.83 | 0.02 |
| 72 | Species_Coprococcus comes | 196.0 | 3.1 | 3.4E-04 | 3.3E-03 | Species | 0.76 | 0.08 |
| 73 | Species_Desulfovibrio piger | 10.8 | 20.6 | 9.5E-07 | 1.2E-05 | Species | 0.17 | 0.41 |
| 74 | Species_Dialister invisus | 108.2 | 6.5 | 1.8E-03 | 1.3E-02 | Species | 1.19 | 0.04 |
| 75 | Species_Faecalibacterium prausnitzii | 3240.0 | 3.4 | 2.9E-06 | 3.5E-05 | Species | 12.24 | 1.57 |
| 76 | Species_Fusobacterium nucleatum | 1054.7 | 4.4 | 8.6E-04 | 7.3E-03 | Species | 4.07 | 0.44 |
| 77 | Species_Lachnospiraceae NK4A136 group bacterium | 328.8 | 8.1 | 2.7E-13 | 9.8E-12 | Species | 1.41 | 0.00 |
| 78 | Species_Ruminococcus bicirculans | 95.7 | 6.1 | 5.8E-04 | 5.3E-03 | Species | 0.36 | 0.02 |
| 79 | Species_Ruminococcus callidus | 10.3 | 21.1 | 6.0E-11 | 1.2E-09 | Species | 0.15 | 0.00 |
| 80 | Species_Selenomonas sputigena | 978.4 | 27.3 | 1.3E-38 | 1.8E-36 | Species | 2.82 | 0.00 |
| 81 | Species_Streptococcus gallolyticus | 104.5 | 8.1 | 1.2E-03 | 9.7E-03 | Species | 0.76 | 0.38 |
| 82 | Species_Streptococcus parasanguinis | 17.1 | 21.8 | 1.7E-13 | 8.1E-12 | Species | 0.11 | 0.00 |

**Table S4: List with discriminant ions from untargeted metabolomics analysis between tumor tissue and tumor-adjacent stroma in the relapse group.** **The following criteria were applied: p-value after FDR correction < 0.0005 and fold change > 2 or < -2).**

| **m/z** | **ion** | **Identification of metabolites** |
| --- | --- | --- |
| \| 408.38533 \| \| --- \| | Not identified | Not identified |
| 778.51726 | [M+Cl]^-^ | PC(13:0/20:2(11Z,14Z)) |
| \| 784.62132 \| \| --- \| | [M-H]^-^ | PC(O-20:0/17:2(9Z,12Z)) |
| \| 794.51143 \| \| --- \| | [M+F]^-^ | PC(14:1(9Z)/22:6(4Z,7Z,10Z,13Z,16Z,19Z)) |
| 807.50031 | [M-H]^-^ | PI(12:0/20:1(11Z)) |
| \| 826.67982 \| \| --- \| | [M-H]^-^ | GlcCer(d18:1(8Z)/24:0(2OH[R])) |
| 833.51611 | [M-H]^-^ | PI(16:0/18:2(9Z,12Z)) |
| 849.54775 | [M-H]^-^ | PI(13:0/22:1(11Z)) |

**Table S5: List with discriminant ions (400 – 1100 m/z) corresponding to lipids from three conditions: 11G5-, 11G5∆*clbQ*-infected HCT116 and non-infected cells.**

| ***m/z*** | **11G5** | **11G5ΔclbQ** | **Non infected** | **Potential lipids** |
| --- | --- | --- | --- | --- |
| 542.45 | **-** | **+** |  | Cer 32:0;O4 |
| 546.45 | **-** | **+** |  | LPC 20:2 |
| 560.45 | **-** | **+** |  | CerP 30:1;O2 ou Cer 36:3;O2 |
| 570.45 | **-** | **+** |  | LPC 22:4 |
| 574.45 | **-** | **+** |  | LPC 22:2 |
| 594.55 | **+** | **-** |  | Cer 38:0;O2 |
| 603.45 |  |  | **+** | [DG (36:10)-H]^-^ |
| 666.45 |  | **+** | **-** | [PE (O-32:5)-H]^-^ |
| 682.55 | **-** | **+** |  | [Cer (t42:0(2OH))-H]^-^ |
| 684.65 |  | **+** | **-** | Not identify |
| 694.45 | **+** |  | **-** | [PE (O-34:5)-H]^-^ |
| 740.55 | **-** |  | **+** | [PE (36:3)-H]^-^ |
| 742.55 | **-** |  | **+** | [PE (36:2)-H]^-^ |
| 800.45 | **+** | **-** |  | [PS (38:9)-H]^-^ |
| 802.55 | **+** |  | **-** | [PC (38:7)-H]^-^ |
| 822.55 | **+** | **-** |  | [PS (O-40:5)-H]^-^ |
| 824.55 | **+** | **-** |  | [PS (O-40:4)-H]^-^ |
| 884.45 | **+** | **-** |  | [PS (44:9)-H]^-^ |
| 888.65 |  | **+** |  | [PS (44:7)-H]^-^ |
| 910.55 | **-** | **+** |  | [PS (42:0)-H]^-^ |
| 913.45 | **+** |  | **-** | [PI (40:4)-H]^-^ |

+ (up-regulated); - (down-regulated)

| ***m/z*** | **11G5** | **11G5ΔclbQ** | **Non infected** | **Potential lipids** |
| --- | --- | --- | --- | --- |
| 680.55 | **-** |  | **+** | [Cer (t42:2(2OH))-H]- |
| 682.55 | **-** | **-** | **+** | [Cer (t42:0(2OH))-H]- |
| 768.55 | **-** |  | **+** | [PE (38:3)-H]- |
| 844.65 | **-** |  | **+** | [PE (44:7)-H]- |

**Table S6: List with discriminant ions (600 – 1100 m/z) corresponding to lipids from three conditions 11G5-, 11G5∆clbQ-infected MC38 and non-infected cells.**

+ (up- regulated); - (down-regulated)
