# Supplementary figures and images for: The colibactin-producing Escherichia coli alters the tumor microenvironment to immunosuppressive lipid overload facilitating colorectal cancer progression and chemoresistance
Source: Gut Microbes. 2024 Feb 28;16(1):2320291. doi: 10.1080/19490976.2024.2320291 (PMC10903627; doi:10.1080/19490976.2024.2320291)

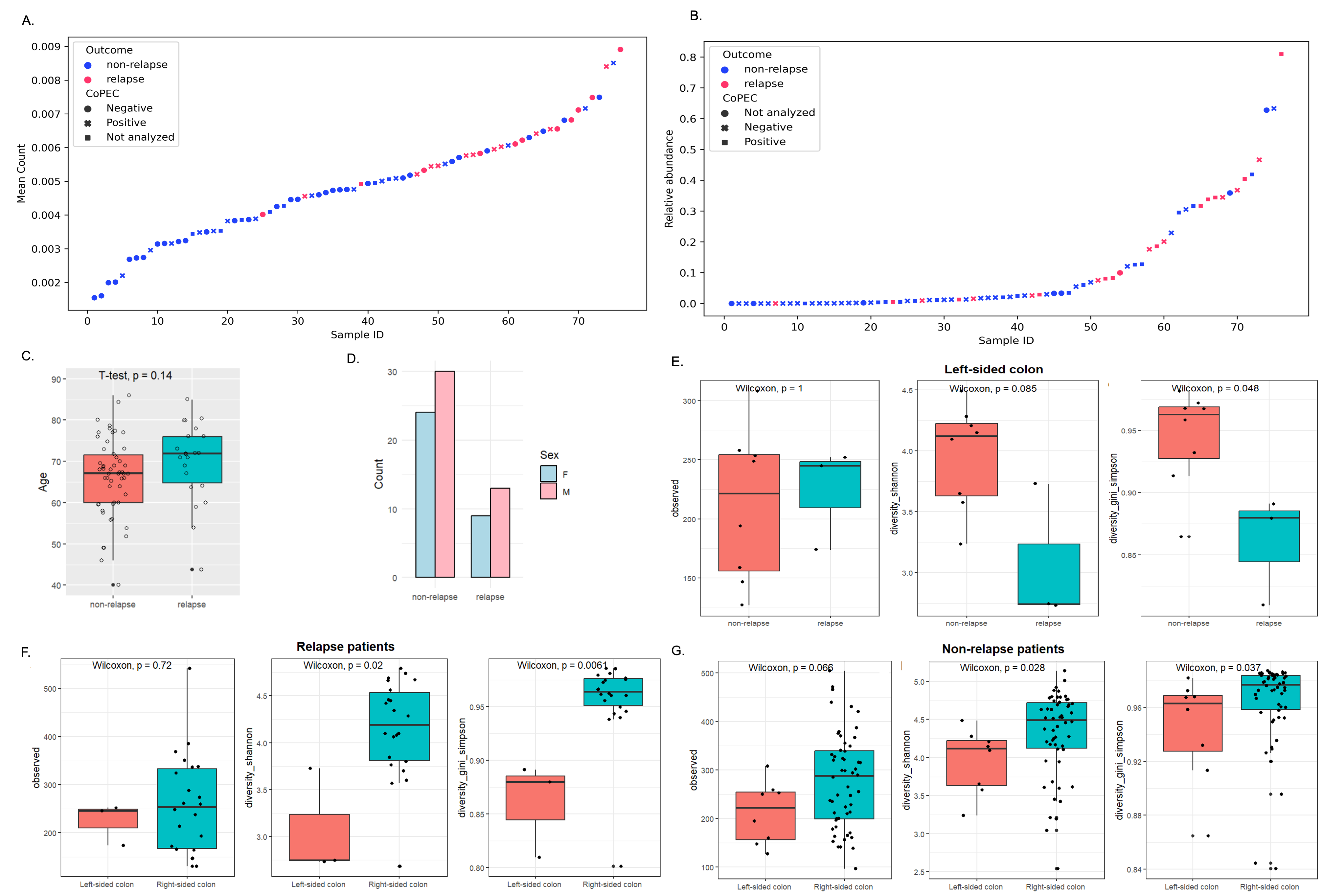

Supplement: Supplemental Material [file KGMI_A_2320291_SM6627.zip › Fig_S1.tiff]

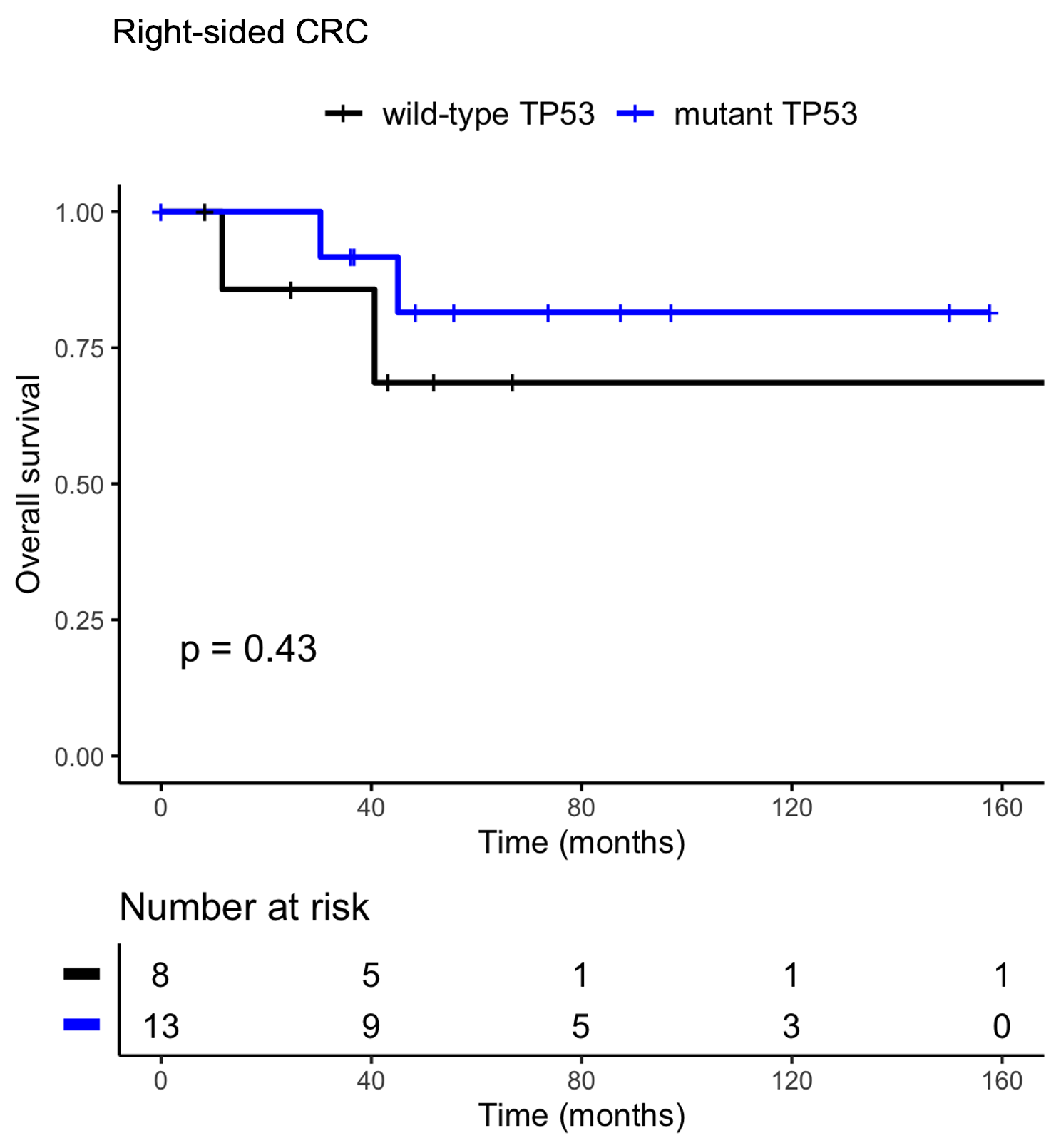

Supplement: Supplemental Material [file KGMI_A_2320291_SM6627.zip › Fig_S2.tiff]

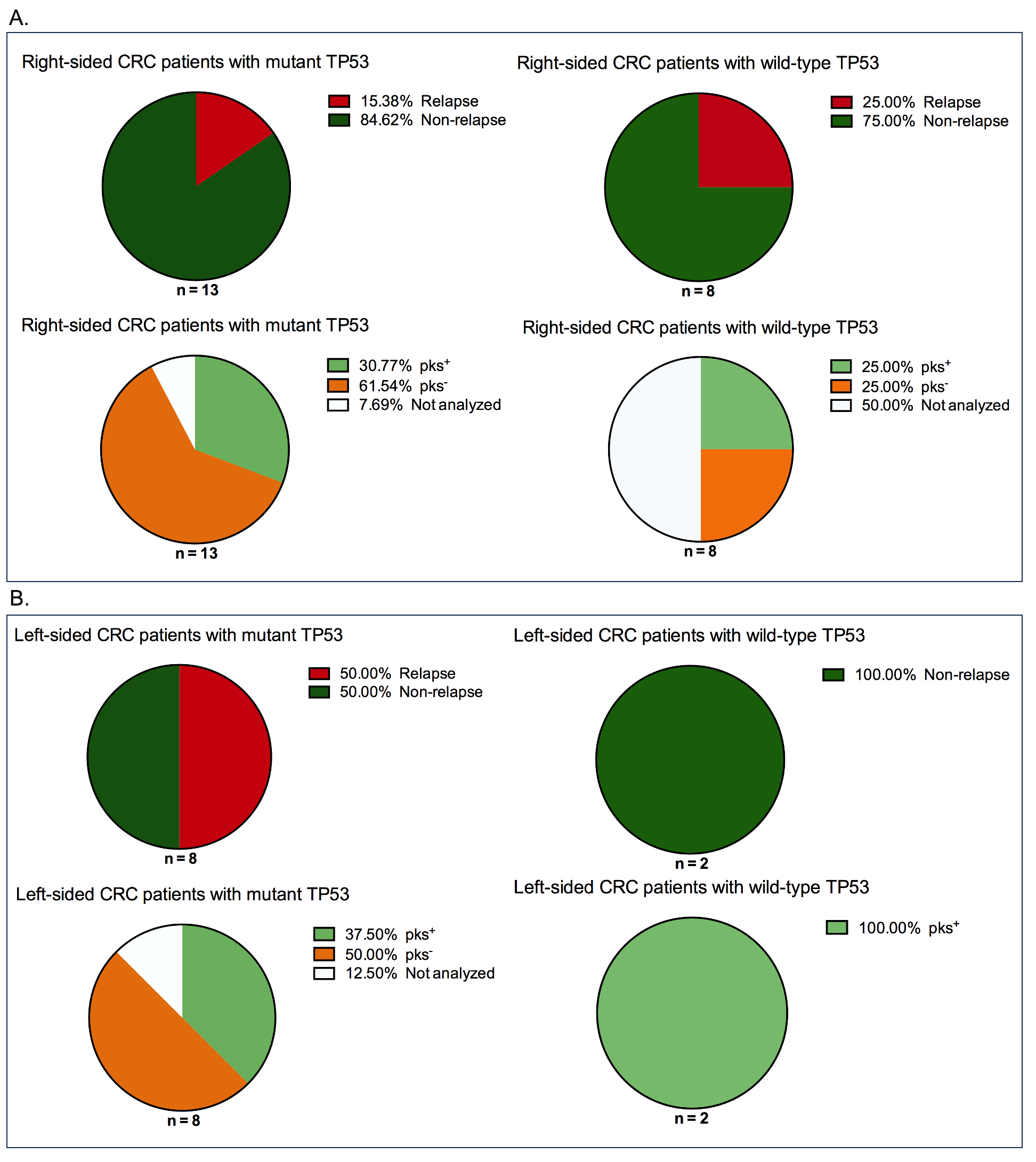

Supplement: Supplemental Material [file KGMI_A_2320291_SM6627.zip › Fig_S3.tif]

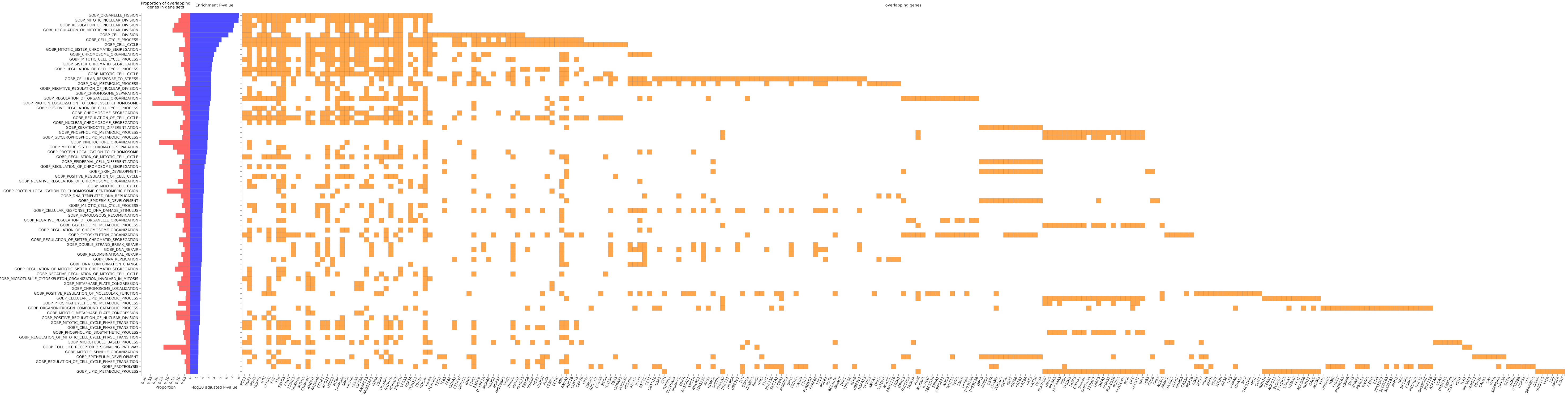

Supplement: Supplemental Material [file KGMI_A_2320291_SM6627.zip › Fig_S4.tiff]

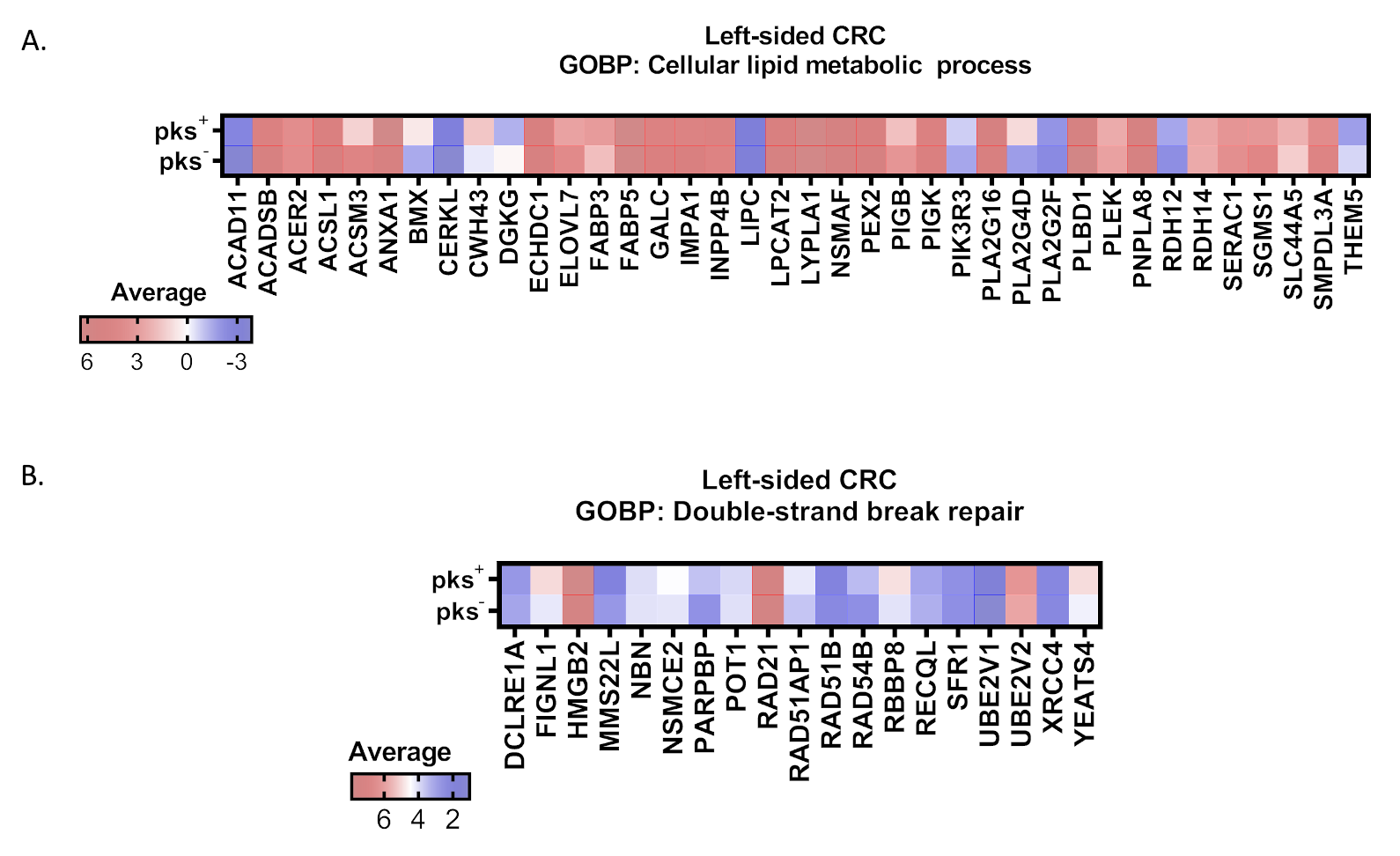

Supplement: Supplemental Material [file KGMI_A_2320291_SM6627.zip › Fig_S5.tiff]

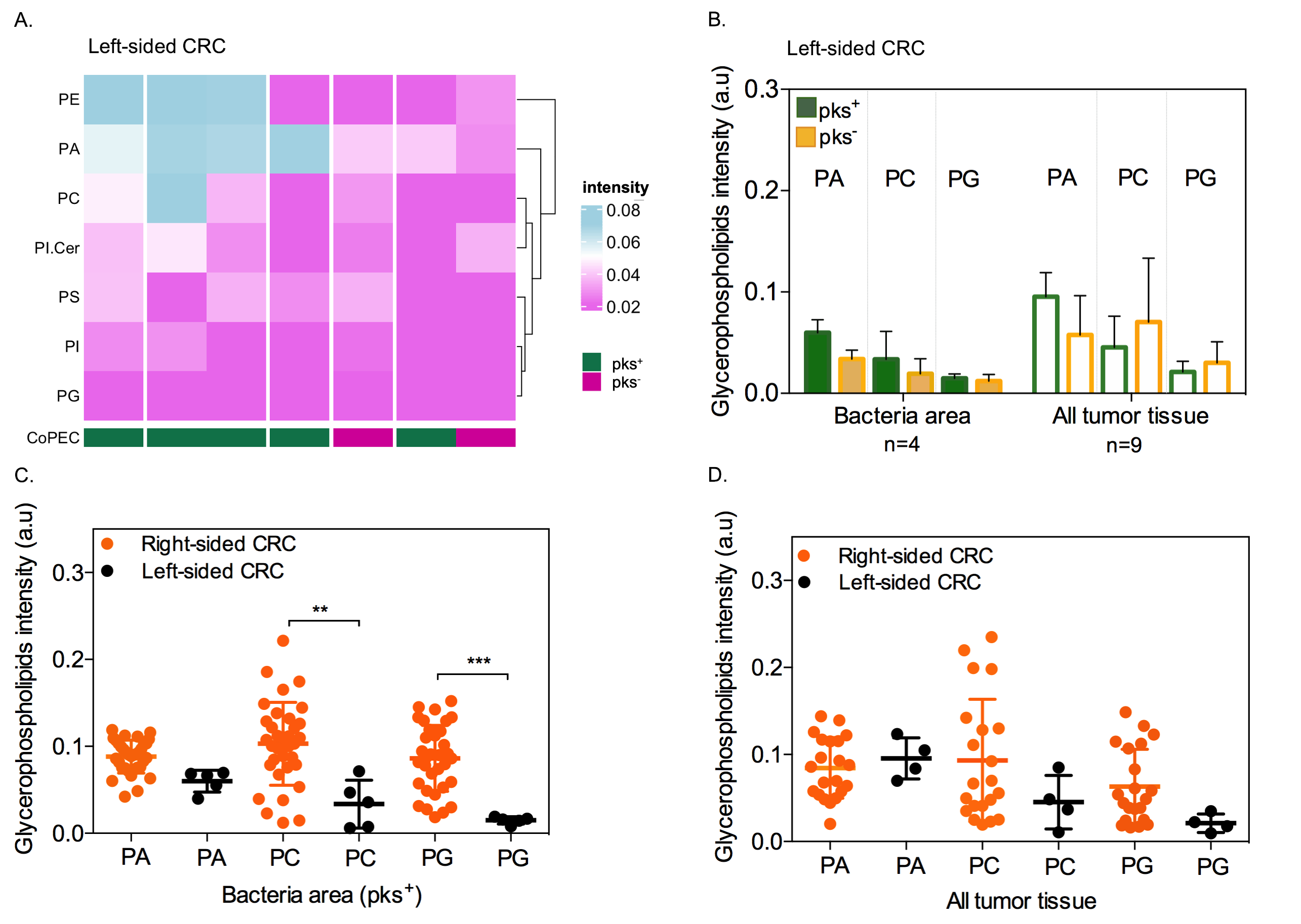

Supplement: Supplemental Material [file KGMI_A_2320291_SM6627.zip › Fig_S6.tiff]

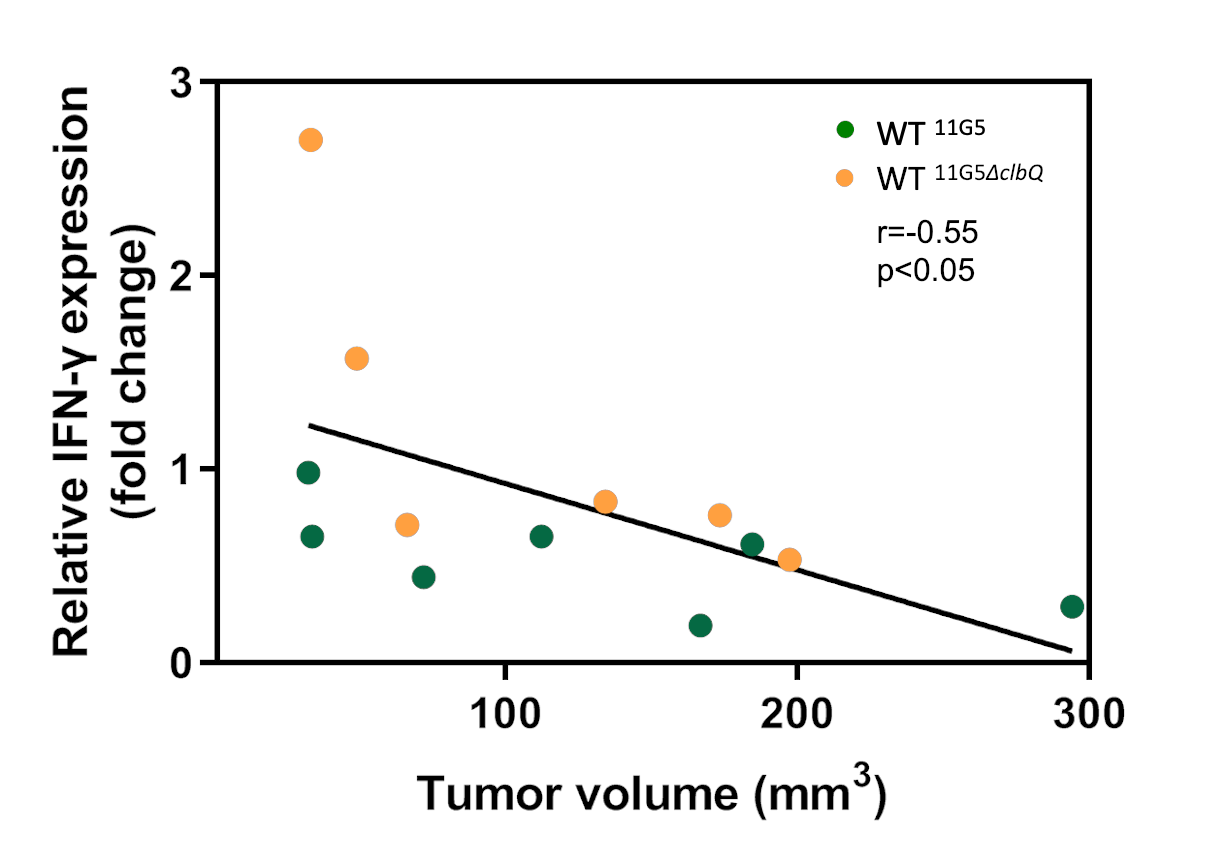

Supplement: Supplemental Material [file KGMI_A_2320291_SM6627.zip › Fig_S7.tiff]

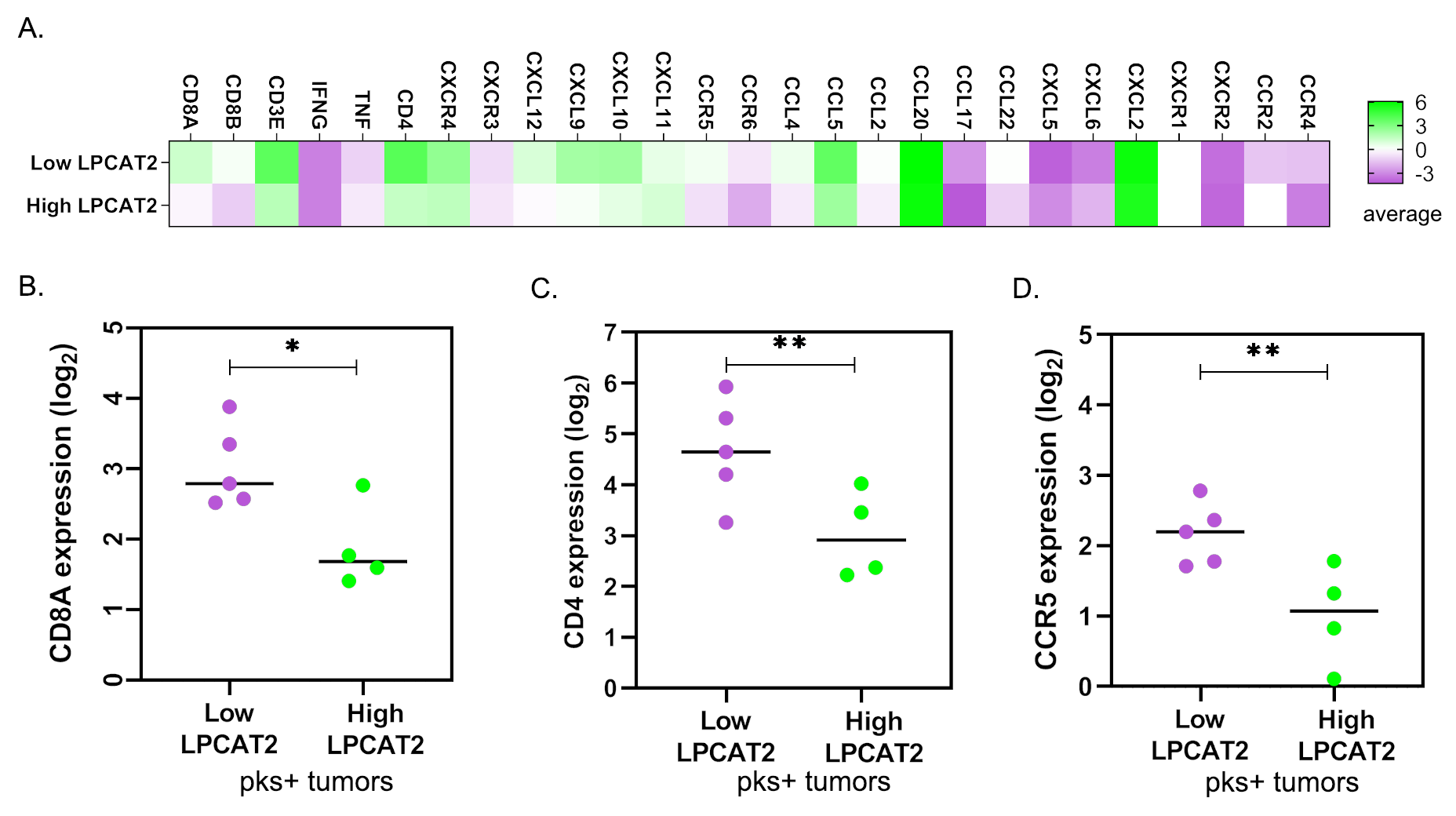

Supplement: Supplemental Material [file KGMI_A_2320291_SM6627.zip › Fig_S8.tiff]

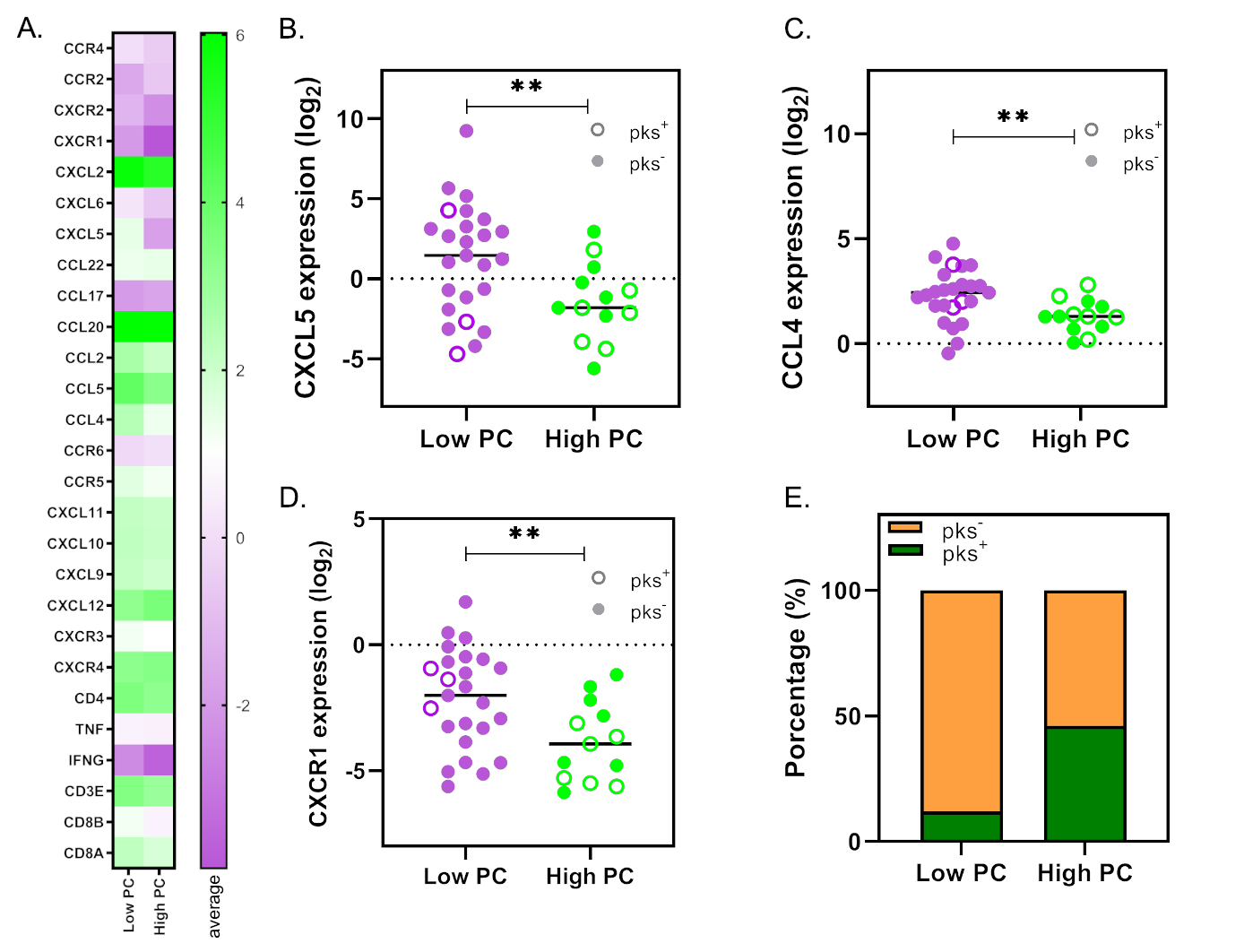

Supplement: Supplemental Material [file KGMI_A_2320291_SM6627.zip › Fig_S9.tiff]
